# The effectiveness of mulligan mobilization with movement (MWM) on outcomes of patients with ankle sprain: a systematic review and meta-analysis

**DOI:** 10.1186/s13102-025-01121-6

**Published:** 2025-04-29

**Authors:** Mohamed M. ElMeligie, Heba A. Abdeen, Hady Atef, Elena Marques-Sule, Rania N. Karkosha

**Affiliations:** 1 Department of Physical Therapy for Basic Sciences, Faculty of Physical Therapy, Ahram Canadian University, Giza, Egypt; 2 Department of Basic Sciences, Faculty of Physical Therapy, Al Hayah University, Cairo, Egypt; 3 Physical Therapy Department for Cardiovascular, Respiratory Disorders and Geriatrics, Faculty of Physical Therapy, Cairo University, Cairo, Egypt; 4 School of Allied Health Professions (SAHP), Keele University, Staffordshire, ST5 5BG UK; 5 Physiotherapy in Motion, Multispeciality Research Group (Ptinmotion), University of Valencia, 46010 Valencia, Spain; 6 Department of Basic Sciences, Faculty of Physical Therapy, Cairo University, Ad Doqi, Giza District, Giza Governorate, 11432 Egypt

**Keywords:** Mobilization with movement, Manual therapy, Mulligan taping, Ankle sprains, Systematic review, Meta-analysis

## Abstract

**Background:**

Ankle sprains are common injuries that cause pain, swelling, and reduced range of motion (ROM), adversely affecting physical activity. In this study, we aim to review the effectiveness of mobilization with movement (MWM) in improving outcomes for patients with ankle sprains.

**Methods:**

We conducted a search of PubMed, Cochrane Library, PEDro, Web of Science, and Scopus up to October 2023 for English trials comparing Mulligan MWM with other treatments. The Cochrane Risk of Bias tool (ROB 2) was used for quality assessment, and mean differences (MD) with 95% confidence intervals (CI) were calculated. Heterogeneity was evaluated using Cochrane’s Q and I^2^ statistics.

**Results:**

A total of 10 trials involving 419 patients (209 in the MWM group and 210 controls) were included. The overall risk of bias was low. MWM significantly reduced pain (MD = - 0.92; 95% CI:[- 1.37, - 0.46]; *P* < 0.0001) and improved ankle ROM (SMD = 1.65; 95% CI:[0.17, 3.14]; *P* = 0.03). MWM also demonstrated superior performance in the Star Excursion Balance Test (SEBT) (MD = 3.15; 95% CI:[1.44, 4.86]; *P* = 0.0003) and Y Balance Test (MD = 4.69; 95% CI:[1.67, 7.70]; *P* = 0.02). However, no significant differences were found in pain pressure threshold (SMD = - 0.10; 95% CI:[- 0.59, 0.39]; *P* = 0.7), stiffness perception (MD = 0.10; 95% CI:[- 0.64, 0.85]; *P* = 0.79), or peroneus longus latency time (MD = - 12.85; 95% CI:[- 22.08, - 3.63]; *P* = 0.006). The quality evaluation showed that the majority of RCTs revealed some concerns, except of two studies that established a low risk of bias. The GRADE assessment classified the overall evidence as low or very low, due to imprecision, risk of bias, and inconsistency.

**Conclusions:**

MWM significantly reduced pain and improved ROM and WBLT scores in patients with ankle sprains. The MWM group also showed enhanced balance in the posterolateral SEBT compared to controls.

**Supplementary Information:**

The online version contains supplementary material available at 10.1186/s13102-025-01121-6.

## Introduction

Ankle sprains rank among the most frequent musculoskeletal injuries globally, occurring at an estimated rate of 2.15 incidents per 1000 individuals daily worldwide [1]. Ankle sprains frequently result in discomfort, inflammation, reduced mobility, and instability, ultimately compromising physical activities and overall quality of life [2, 3]. Considering the high prevalence and significant impact of ankle sprains, prioritizing the discovery of efficacious treatments is imperative [4].

Ligament damage frequently accompanies ankle sprains, with a notable impact on the anterior talofibular and calcaneofibular ligaments [5]. Ankle sprains often lead to functional instability, contributing to negative consequences such as reduced proprioception, compromised neuromuscular control, weakened muscle strength, and impaired postural stability [6].

Ankle sprains are typically managed with conservative approaches, which have evolved over time. Historically, the PRICE (Protection, Rest, Ice, Compression, Elevation) principle was widely recommended for managing soft tissue injuries, including ankle sprains. However, recent evidence suggests a shift toward more comprehensive rehabilitation strategies, emphasizing tissue healing, early mobilization, and patient-centered care [7].

According the American family physician, it is common to advise RICE (rest, ice, compression, elevation) for treating ankle sprains [8]; however, they do not have a direct impact on improving range of motion (ROM) or functional outcomes. Although these conservative strategies are crucial for alleviating pain and swelling linked to ankle sprains by fostering tissue repair and mitigating inflammation, their focus primarily revolves around symptom management rather than directly targeting rehabilitative goals [9]. Hence, exploring supplementary therapeutic methods aimed at improving ROM and functionality should be contemplated to maximize long-term rehabilitation outcomes [10].

Mobilization with Movement (MWM) is a manual technique that entails applying continuous gliding or rhythmic movements to targeted joint structures while the patient actively engages in controlled manner [11]. Physical therapists employ MWM techniques to accelerate recovery [12], operating on the principle that integrating joint mobilization with active movement stimulates mechanoreceptors, alleviates pain, and enhances mobility [13, 14]. Moreover, MWM for ankle sprains focuses on restoring dorsiflexion ROM and posterior talar glide, as these are frequently observed issues in chronic ankle sprains [15].

However, a previous review done by [16] [16] found mixed results regarding the effects of MWM for ankle sprains, Weerasekara et al. encountered several limitations, such as a limited number of studies, a narrow scope of outcomes, uncertain bias risk, and absence of quality assessment.

Our current study aims to offer a comprehensive and refined evaluation of the evidence by incorporating recent studies assessing additional outcomes and demonstrating low bias risk. We added other additional outcomes, peroneal latency time, the delay in peroneus longus muscle activation after ankle inversion, is a key factor in ankle stability.

The newer PEACE & LOVE framework has gained recognition, as it not only addresses acute injury management (PEACE: Protection, Elevation, Avoidance of anti-inflammatories, Compression, Education) but also incorporates long-term rehabilitation strategies (LOVE: Load, Optimism, Vascularization, Exercise) that enhance tissue repair and functional recovery [7].

Exercise therapy plays a critical role in the recovery from ankle sprains, significantly reducing the risk of re-injury and improving both clinical outcomes and patient-reported outcome measures (PROMs) [17]. Meta-analyses have shown that rehabilitation protocols involving progressive loading, balance training, and functional exercises contribute to better long-term outcomes in patients with ankle sprains. Incorporating exercise therapy early in the rehabilitation process not only enhances recovery but also supports a faster return to physical activity while minimizing the risk of chronic instability and recurrent sprains [18]​.

Additionally, establishing whether MWM results in substantial enhancements in outcomes like pain reduction, increased ROM, and resumption of activity holds paramount importance. That would offer valuable guidance for refining ankle sprain rehabilitation protocols, potentially enhancing patient recovery, curbing healthcare expenses associated with prolonged recovery or recurrent injuries, and reducing downtime from work or physical activities [19]. Considering the high prevalence of ankle sprains, even small improvements in outcomes could have a meaningful impact at a population level [20].

This systematic review aims to assess the effectiveness of MWM on essential outcomes, thereby determining its suitability as an evidence-based recommendation for ankle sprain rehabilitation and offering clinicians valuable guidance regarding optimal treatment protocols.

## Materials and Methods

This systematic review was conducted according to the Guidelines of Cochrane Handbook for Systematic Reviews of Interventions [21], and reported following the Preferred Reporting Items for Systematic Reviews and Meta-Analyses (PRISMA) guidelines [22]. Meta-analysis was performed for data synthesis where appropriate. This study's protocol was prospectively registered on PROSPERO with registration number CRD42022345022.

### Search Strategy

We searched the following databases: Cochrane, PubMed, Physiotherapy Evidence Database (PEDro), Web of Science (WOS), and Scopus from the inception until October 2023. Our search was restricted to English articles. Our search strategy included terms related to the Mulligan concept and ankle mobilization. We used the following search strategy: ((Mulligan*) OR (Mulligan mobilization) OR (Mulligan concept) OR (Mulligan method) OR (Mulligan technique) OR (mobilization with movement) OR (MWM)) AND ((ankle sprain) OR (ankle injuries) OR (ankle instability) OR (lateral ankle sprain) OR (chronic ankle instability) OR (acute ankle sprain)).

### Eligibility Criteria

We have prespecified our PICOS criteria (Population, intervention, comparator, outcomes, and study design) prior to screening as follows: P (Population): We included studies involving adult individuals diagnosed with ankle sprain either inversion or eversion. I (Intervention): The intervention utilized was MWM (A technique that involves the application of sustained passive movement to a joint while the patient actively performs previously painful or limited movements). C (Comparator): The comparators included various groups like placebo interventions (sham mobilization), any non-MWM mobilization such as osteopathic or Maitland mobilization methods, or electrotherapy. O (Outcomes): The primary outcomes included pain scores and ankle joint ROM, while secondary outcomes included pain pressure threshold, balance capabilities, and weight-bearing lunge test (WBLT). S (Study design): To achieve the most solid quality of evidence, we restricted the study design to only randomized controlled trials (RCTs). Exclusion criteria included any different study design rather than RCTs (single arm studies, cohorts, case controls, thesis, and conference abstracts) and non-English studies.

### Study selection

Using Endnote software, two independent reviewers (HA and RN) collected the different records from the databases and removed duplicates using RAYYAN software. The retrieved references were screened to assess their relevance. The screening was done in two steps; title and abstract screening, followed by full-text screening for final eligibility. Disagreements were settled through conversations with the third author (EM).

### Quality assessment

The Cochrane Risk of Bias II (ROB II) tool [23] was used to assess the quality of the included randomized controlled trials (RCTs) by two independent reviewers (ME and HAA). The ROB II tool evaluates bias across five domains: the randomization process, deviations from intended interventions, missing outcome data, measurement of the outcome, and selection of the reported result. Each study was categorized as having either “low risk,” “some concerns,” or “high risk” of bias based on these domains. Any disagreements between the reviewers were resolved through discussion and consensus with a senior author (RN).

In addition, to evaluate the overall quality of evidence, we employed the GRADE (Grades of Recommendation, Assessment, Development, and Evaluation) approach [24]. Eligible studies were assessed for risk of bias, imprecision, inconsistency, indirectness, publication bias, and other relevant factors. The overall quality of evidence was classified as high, moderate, low, or very low for each outcome. Any disagreements between the two reviewers (ME and EM) were resolved through discussion, with input from a third reviewer (HAA) when necessary.

Missing data were not recovered through direct contact with the original authors but were addressed through the risk of bias assessment using the ROB2 tool and considered in the GRADE evaluation.

### Data Extraction

Data extraction was performed independently by two authors (HA and RN) and recorded in an Excel spreadsheet. The extracted data included: [1] baseline characteristics and demographic details of the included populations, [2] outcome measures including pain scores, ROM, Star Excursion Balance Test (SEBT), balance assessments, WBLT, peroneal latency (PL) time, and perceptions of stiffness. Risk of bias (ROB) was assessed for all included studies in accordance with Cochrane ROB 2 tool.

### Data Synthesis

Statistical analysis was performed using Review Manager Software (RevMan 5.4.1 for windows). Since all outcomes were continuous, we reported results as mean difference (MD) or standardized mean difference (SMD) with 95% confidence intervals (CIs), allowing comparison across studies using different measurement scales. MD was used when studies reported outcomes on the same scale, such as pain (VAS) and ROM. SMD was used when outcomes were measured on different scales across studies, such as the SEBT. The inverse variance method was applied for weighting. We assessed heterogeneity using chi-square and I^2^ tests. If heterogeneity was significant (*P* < 0.1, I^2^ > 50%), a random-effects model was used; otherwise, a fixed-effects model was applied.

## Results

### Literature search

**Fig. 1:**
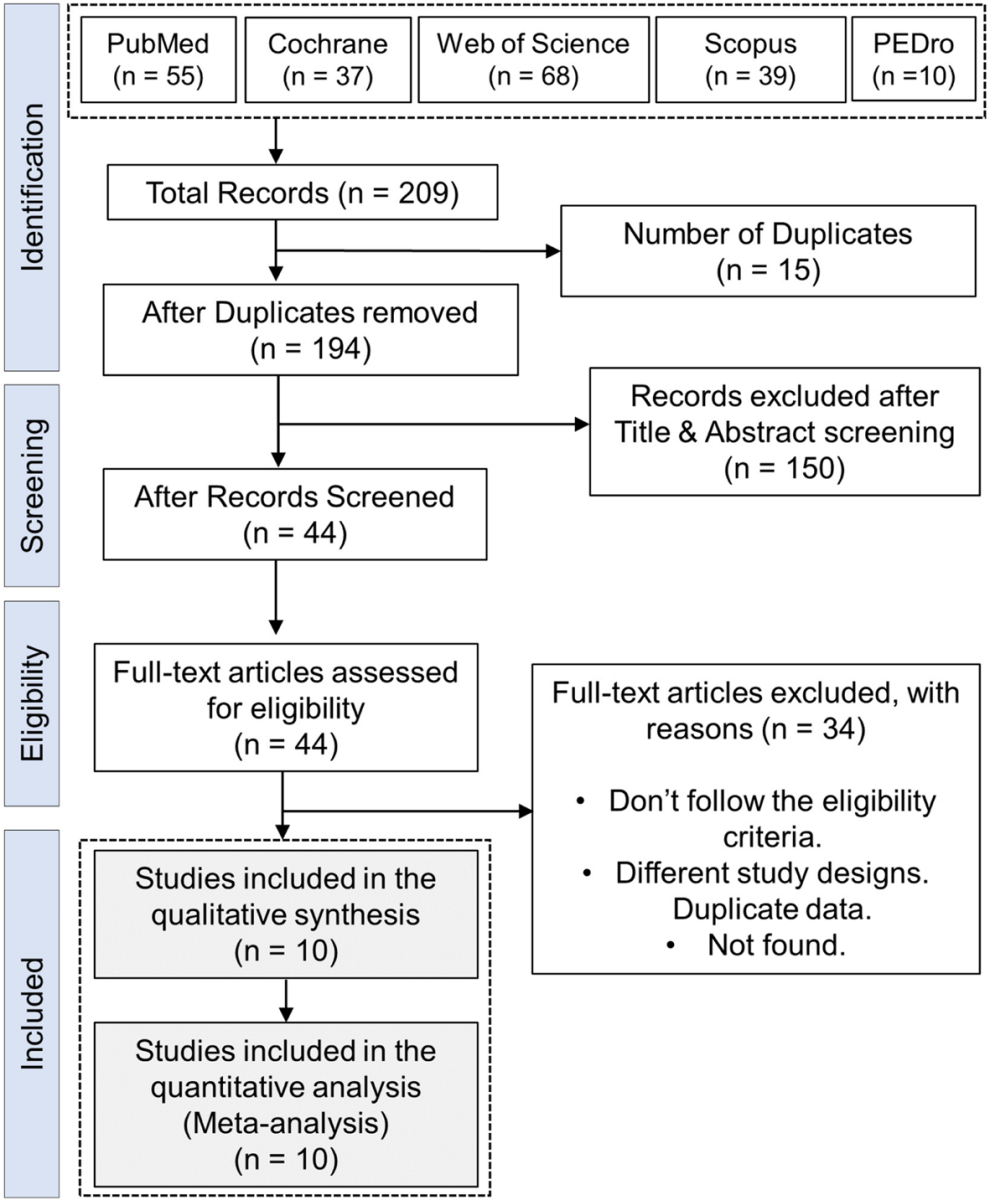
PRISMA flow diagram of the literature search results

We included ten trials [14, 25–33] beginning with 209 records retrieved from five databases. After removing duplicates, 194 records remained for screening, during which 150 were excluded based on title and abstract assessments, leaving 44 for eligibility evaluation. Of these, 34 were excluded for reasons such as not meeting eligibility criteria or different study designs. The flow of the study selection process is illustrated in the PRISMA flow diagram in Fig. 1.

A total of 419 patients were enrolled in this meta-analysis. The Mulligan group included 209 cases, and the control group involved 210 patients. The mean age of the patients in the Mulligan was 26.46 years while in the control group was 27.54 years.

### Characteristics of the included studies

Tables 1, 2, and 3 present a summary of the included trials, including demographic data, baseline characteristics, pain scores, and symptom duration of the participants. Table 1 demonstrates an overview of the demographic characteristics of the participants included in this review. Table 2 illustrates the baseline characteristics of these participants data analyzed in the review. Table 3 provides data for both the participants pain scores and symptom duration at baseline, aiding in understanding their conditions before any interventions noted within this study.

**Table 1:** shows the demographic data of the included patients. SD; standard deviation, NA; non-available, BMI; body mass index

Study ID	Country	Study design	Sample size		Age; years mean (SD)		Males		Females		BMI, mean (SD)	
			Mulligan	Placebo	Mulligan	Placebo	Mulligan	Placebo	Mulligan	Placebo	Mulligan	Placebo
**Collins 2004**	Australia	Double-blinded RCT (cross-over)	16	16	28.25 ± 9.3	28.25 ± 9.3	8	8	8	8	NA	NA
**Gogate 2020**	India	Randomized controlled trial	16	16	26.1 ± 6.6	28.4 ± 7.0	9	10	7	6	NA	NA
**Nguyen 2021**	Belgium	Pragmatic &RCT	21	22	22.6 ± 3.7	22.1 ± 2.12	8	8	13	14	NA	NA
**Norouzi 2021**	Iran	Double blinded RCT	20	20	29.12 ± 7.50	33.29 ± 5.75	10	8	6	9	26.12 ± 3.7	27.00 ± 3.5
**Phong Nguyen 2020**	Belgium	RCT	25	24	23 ± 2.5	21.7 ± 1.74	19	17	6	7	NA	NA
**Simsek 2018**	Turkey	RCT (cross over)	26	26	28.9 ± 5.7	28.9 ± 5.7	16	16	10	10	21.6 ± 1.2	21.6 ± 1.2
**Alves 2018**	Portugal	Randomized, placebo-controlled, crossover trial	16	16	21.5 ± 2.8	21.5 ± 2.8	10	10	6	6	24.1 ± 2.6	24.1 ± 2.6
**Cruz-Diaz 2014**	Spain	Randomized, double-blind, placebo-controlled trial	30	31	26.83 ± 4.62	29.55 ± 9.44	17	17	13	14	23.57 ± 2.88	23.19 ± 2.15
**Reid 2007**	Canada	Randomized controlled crossover trial	23	23	25 ± 9	25 ± 9	8	8	15	15	NA	NA
**Shadegani 2023**	Iran	Randomized, single blind crossover trial	16	16	29.37 ± 6.08	29.37 ± 6.08	5	5	11	11	NA	NA

**Table 2:** shows the baseline characteristic of the included participant

Study ID	Weight (kg) (SD)		Height (cm) (SD)		Affected side				ROM (cm) (SD)		WBLT distance (cm) (SD)	
					Right		Left					
	Mulligan	Placebo	Mulligan	Placebo	Mulligan	Placebo	Mulligan	Placebo	Mulligan	Placebo	Mulligan	Placebo
**Collins 2004**	NA	NA	NA	NA	NA	NA	NA	NA	NA	NA	NA	NA
**Gogate 2020**	70.2 ± 9.2	70.4 ± 9.6	170.2 ± 7.6	169.9 ± 5.6	8	8	8	8	26.7 ± 6.8	23.3 ± 4.8	NA	NA
**Nguyen 2021**	NA	NA	NA	NA	12	14	9	8	NA	NA	8.6 ± 2.72	9.2 ± 2.78
**Norouzi 2021**	75.82 ± 12.2	79.05 ± 10.7	170.75 ± 8.1	171.17 ± 8.5	NA	NA	NA	NA	4.93 ± 1.18	4.83 ± 1.7	NA	NA
**Phong Nguyen 2020**	70.2 ± 10.5	71.5 ± 11.3	176.4 ± 9.2	177.9 ± 8.9	NA	NA	NA	NA	NA	NA	11.6 ± 3.33	12.5 ± 3.33
**Simsek 2018**	NA	NA	NA	NA	NA	NA	NA	NA	NA	NA	NA	NA
**Alves 2018**	77.2 ± 14.1	77.2 ± 14.1	178 ± 0.13	178 ± 0.13	5	5	11	11	NA	NA	NA	NA
**Cruz-Diaz 2014**	NA	NA	1.71 ± 0.09	1.72 ± 0.07	21	20	9	11	53.99 ± 1.58	53.72 ± 1.68	NA	NA
**Reid 2007**	69 ± 11	69 ± 11	170 ± 9	170 ± 9	NA	NA	NA	NA	9.92 ± 3.85	10.14 ± 3.87	NA	NA
**Shadegani 2023**	67.62 ± 12.95	67.62 ± 12.95	167.25 ± 2.29	167.25 ± 2.29	NA	NA	NA	NA	NA	NA	NA	NA

*SD* standard deviation, *NA* non-available, *WBLT* weight-bearing lunge test, *ROM* range of motion

**Table 3:** shows the baseline pain score and duration of symptoms

Study ID	Pain (VAS)or (NRS)		Symptom duration (months)		Condition being studied	Intervention characteristics
	Mulligan	Placebo	Mulligan	Placebo		
**Collins 2004**	NA	NA	NA	NA	subacute ankle sprains	Mulligan’s MWM
**Gogate 2020**	5.9 ± 0.6	5.9 ± 0.6	NA	NA	grade I and II inversion ankle sprain	mobilization with movement, manual therapy
**Nguyen 2021**	2.4 ± 1.49	1.9 ± 1.66	2 ± 1.69	1.8 ± 1.61	lateral ankle sprains (Grade I–II)	(MWM) or a sham
**Norouzi 2021**	5.43 ± 1.26	6.00 ± 1.7	NA	NA	grade two lateral ankle sprain	Maitland's mobilization & Mulligan's mobilization
**Phong Nguyen 2020**	1.7 ± 1.49	1.1 ± 1.18	6 months	NA	Ankle injuries including ankle sprain	ITFMWM on the restricted and painful ankle
**Simsek 2018**	2.93 ± 1.2	2.93 ± 1.2	NA	NA	Chronic Ankle Instability	Mulligan distal fibular taping technique
**Alves 2018**	NA	NA	NA	NA	Chronic ankle instability	Mulligan fibular repositioning taping vs placebo taping
**Cruz-Diaz 2014**	NA	NA	NA	NA	Chronic ankle instability	Mulligan mobilization with movement vs sham mobilization vs control
**Reid 2007**	NA	NA	24	24	Chronic ankle instability	Mulligan mobilization with movement vs sham
**Shadegani 2023**	NA	NA	14.29 ± 7.31	14.29 ± 7.31	Chronic ankle instability	Kinesio taping vs Mulligan taping

*SD* standard deviation, *NA* non-available, mobilization with movement, *ITFMWM* inferior tibiofibular mobilization with movement

### Risk of Bias Assessment

**Fig. 2:**
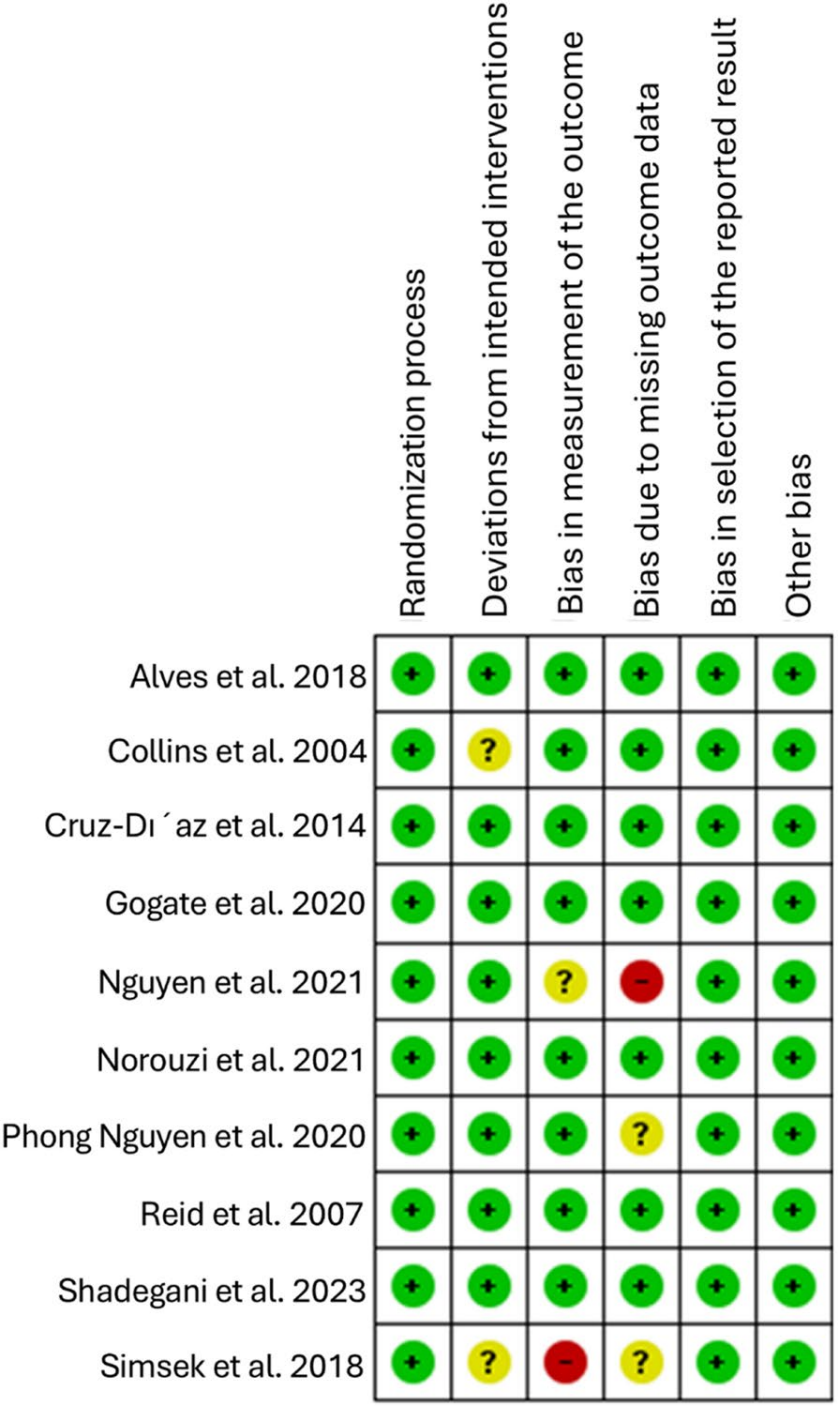
Risk of bias assessment (ROB 2) summary of the included studies

The quality of the included studies revealed an overall “some concern” risk of bias except for two studies, Norouzi 2021[25] and Shadegani 2023 [32], with an overall “low” risk of bias. Regarding domains of deviations from intended interventions, missing outcome data, and measurement of the outcome, most studies indicated some concern risk of bias. Concerns were identified in the randomization process domain for Simsek (2018) [26], Nguyen (2021)[28], Collins (2004) [29], Alves (2017) [30], and Reid (2007) [33]. The absence of protocol registration numbers for multiple studies raised concerns in the fifth domain regarding the selection of reported results. Figure 2 presents a summary of the risk of bias assessment. The ROB assessment for each outcome of interest revealed that all outcomes were rated as “Some Concern,” except for Ankle ROM in Norouzi (2021) [25] and Peroneus Longus Latency Time in Shadegani (2023) [32], both of which exhibited a low risk of bias. The outcomes of interest for the ROB assessment are detailed in **supplementary file 1**.

### Outcomes

#### Pain (measured by Visual Analogue Scale)

**Fig. 3:**
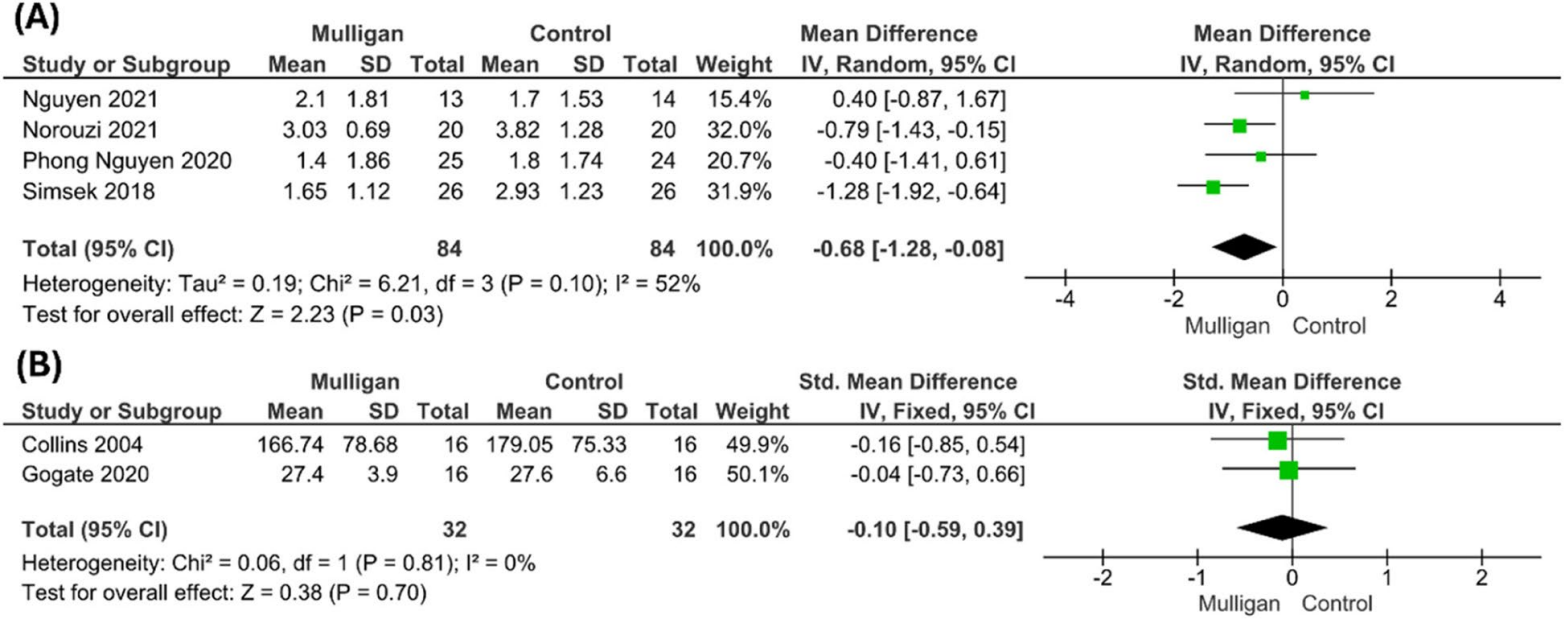
Forest plot of mean difference (MD) in **(a)** visual analogue scale **(b)** standardized mean difference (SMD) in pain pressure threshold

The pooled meta-analysis of four studies [25–28] VAS assessment in 168 participants showed a significant pain reduction in Mulligan group compared to control (MD = − 0.68; 95%CI: [− 1.28, − 0.08], P = 0.03). The pooled studies were heterogeneous (P = 0.1; I^2^ = 52%) and the heterogeneity was best resolved by excluding [27, 28] ((P = 0.3,I^2^ = 17%) and the results remained significant favoring Mulligan over control (MD = − 0.92; 95% CI:[− 1.37, − 0.46]; *P* < 0.0001). Figure 3a***.***

#### Pain Pressure threshold

The meta-analysis results of two studies [14, 29] showed no significant difference between both Mulligan group and control group (SMD = − 0.10; 95% CI: [− 0.59, 0.39]; P = 0.7). The pooled studies were homogenous, and no heterogeneity detected between the pooled studies (P = 0.81; I^2^ = 0). Figure 3b***.***

#### Ankle range of motion (ROM)

**Fig. 4:**
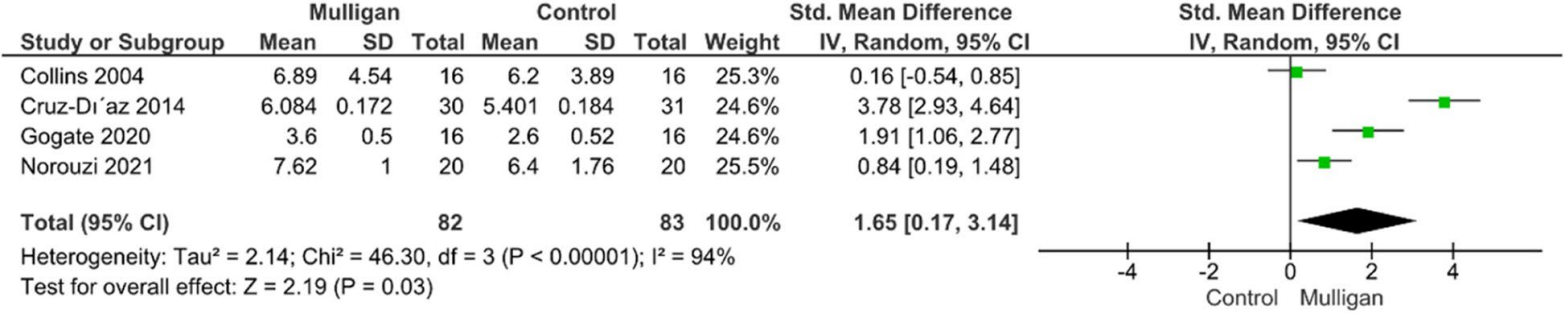
Forest plot of standardized mean difference (SMD) in ankle range of motion (ROM)

Four of the included trials reported data concerning ROM [14, 25, 29, 31]. The meta-analysis results showed a significantly higher ROM improvement in Mulligan group compared to controls (SMD = 1.65; 95% CI: [0.17, 3.14]; P = 0.03). The pooled studies were heterogeneous (*P* < 0.0001, I^2^ = 94%) and the heterogeneity could not be resolved by leave one out test due to high variation between the included studies mostly due to difference in control group intervention between placebo and Maitland. To overcome the heterogeneity, the analysis was done using random effect model. Figure 4***.***

#### Star excursion balance test (SEBT)

**Fig. 5:**
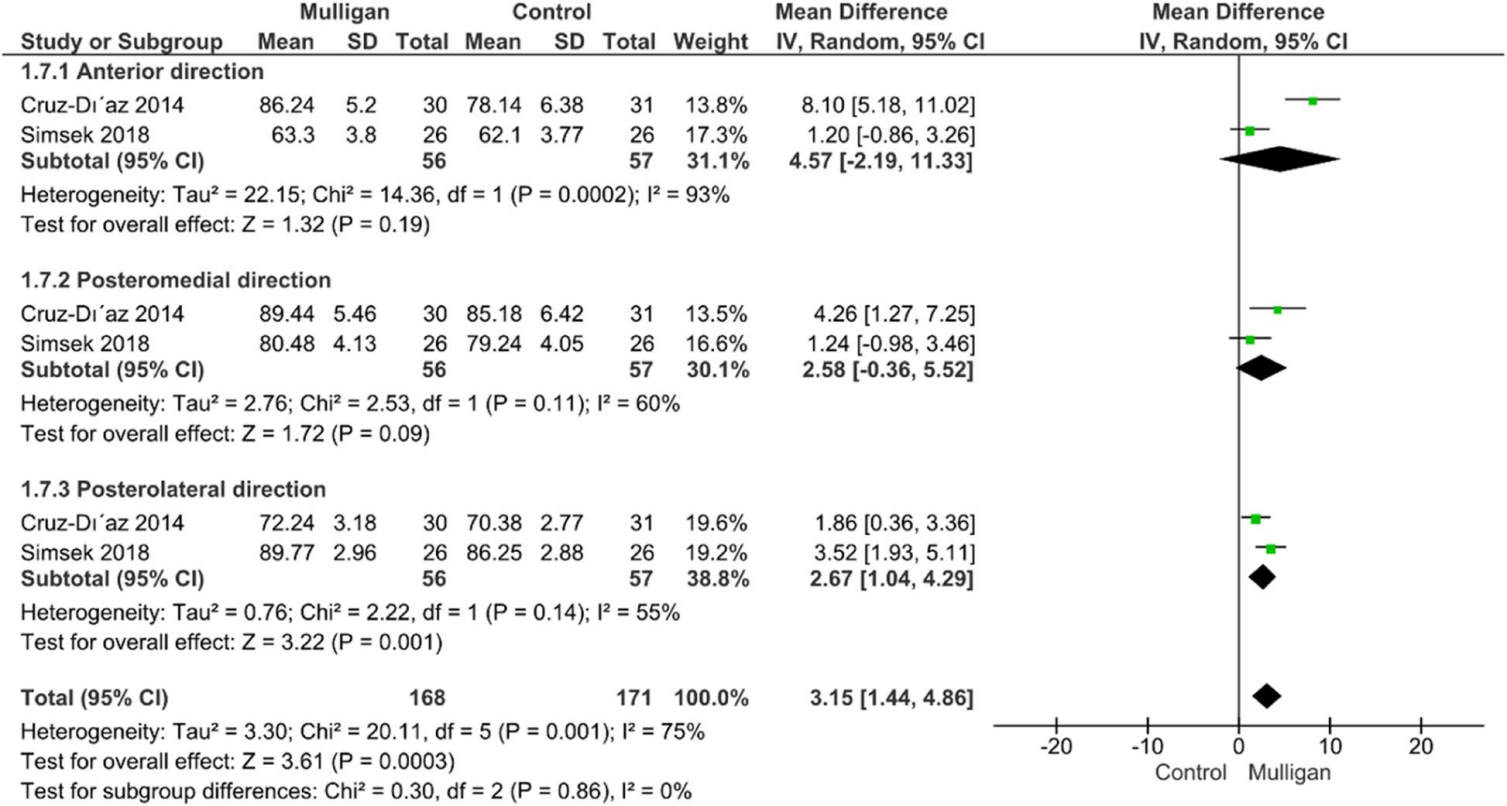
Forest plot of standardized mean difference (SMD) in star excursion balance test

The star excursion balance test (SEBT) was reported by Cruz-Díaz [31] et and Simsek et al. [26] in three directions (Anterior, posteromedial, and posterolateral). Each direction was presented in a different subgroup. The analysis showed a significant improvement in Mulligan group rather than control regarding the posterolateral direction (MD = 2.67 [1.04, 4.29], P = 0.01); however, no significant difference between either anterior or anteromedial direction. Notably, the overall meta-analysis of star excursion balance test favoured Mulligan group (MD = 3.15; 95% CI:[1.44, 4.86], P = 0.0003). The pooled studies were heterogenous (P = 0.001, I2 = 75%) and the heterogeneity could not be resolved due to the limited number of the included studies. Figure 5***.***

#### Y balance test

**Fig. 6:**
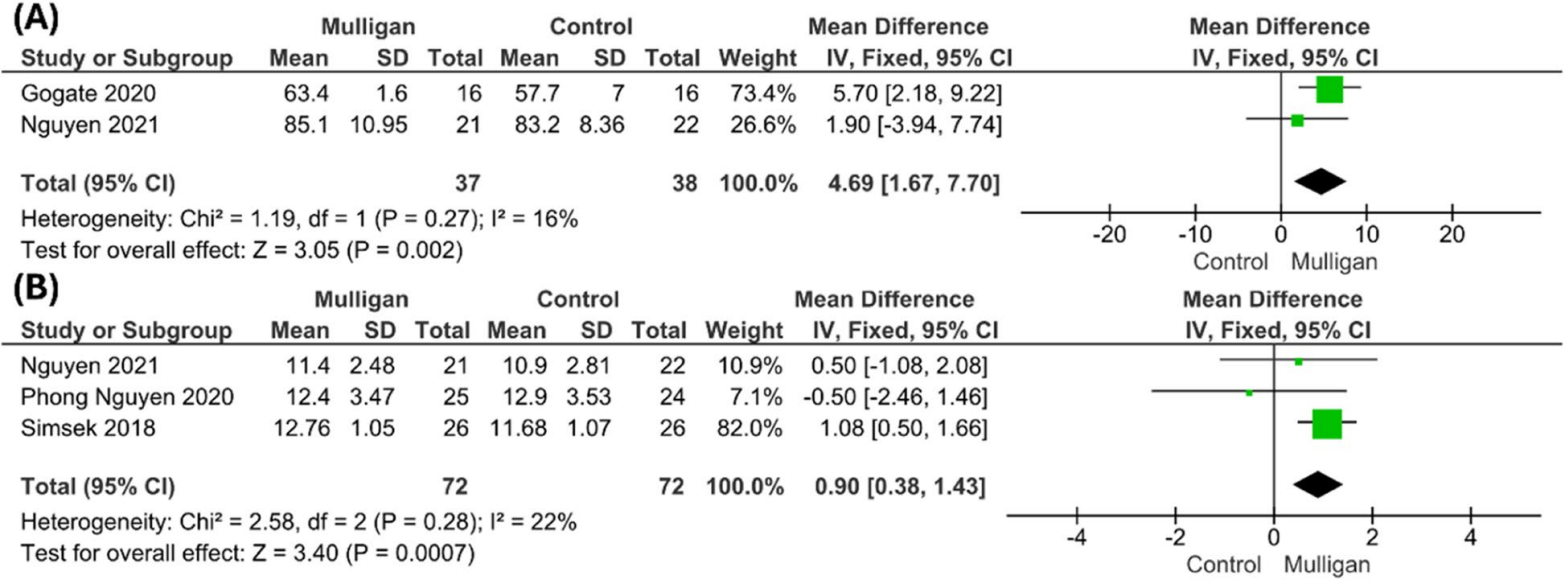
Forest plot of standardized mean difference (MD) in **(a)** Y balance test **(b)** weight bearing lunge test (WBLT)

Two studies [14, 28] reported that Y balance test (YBT) and the analysis favoured the MWM group significantly over the control group (MD = 4.69; 95% CI:[1.67, 7.70]; P = 0.02). The pooled studies were homogenous (P = 0.27; I^2^ = 16%). Figure 6a***.***

#### Weight Bearing Lunge test (WBLT)

The pooled analysis of three studies [26–28, 33] that reported WBLT scores showed significant improvement in mulligan’s mobilization group compared to controls (MD = 0.90; 95% CI: [0.38, 1.43]; P = 0.0007). The pooled studies were homogenous and no significant was heterogeneity detected between the pooled studies (P = 0.28; I^2^ = 22%) Fig. 6b

#### Stiffness perception

**Fig. 7:**
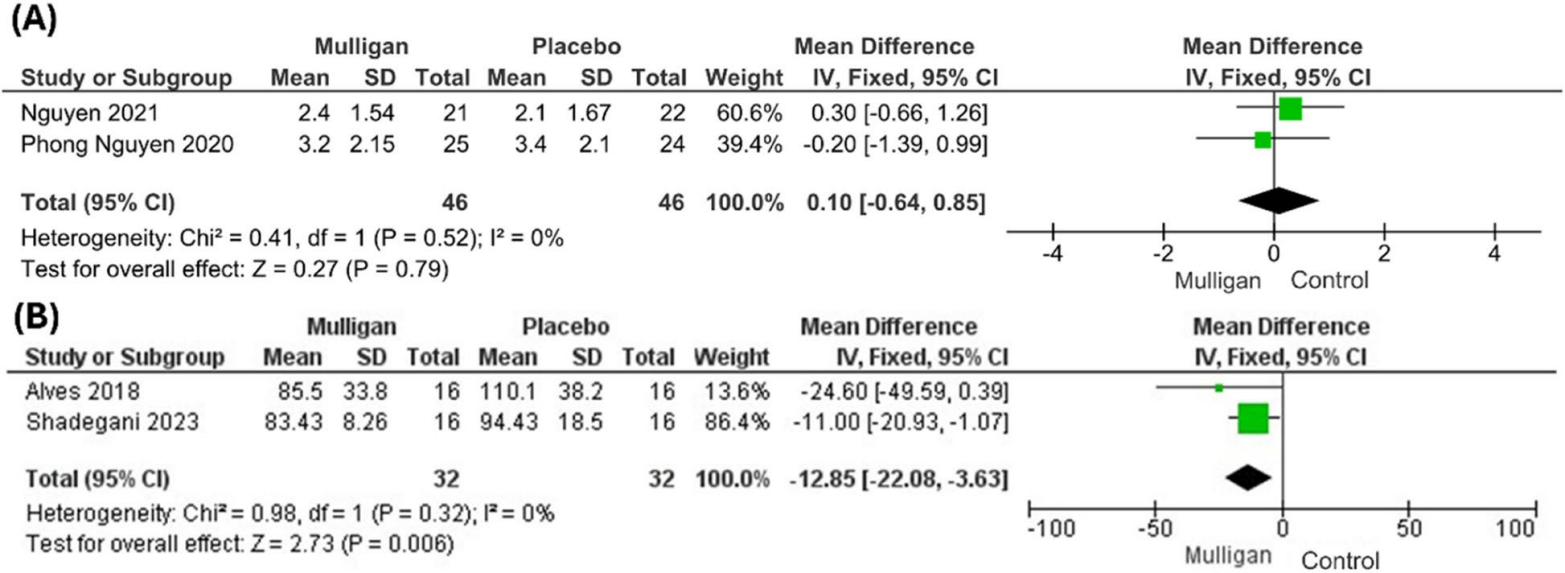
Forest standardized of mean difference (MD) in **(a)** stiffness perception **(b)** peroneus longus latency time

Data about stiffness perception were retrieved from two studies [27, 28]. There was no significant difference between both groups regarding stiffness perception (MD = 0.10; 95% CI: [− 0.64, 0.85]; P = 0.79). The pooled studies were homogenous (P = 0.52; I^2^ = 0%). Figure 7a***.***

#### Peroneus longus latency time

The outcome was reported by two studies [30, 32]. The pooled analysis showed that the latency time significantly decreased in the MWM group compared to controls (MD = − 12.85; 95% CI: [− 22.08, − 3.63], P = 0.006)). The pooled studies were homogenous (P = 0.32); I^2^ = 0%). Figure 7b*.*

### Qualitative synthesis

#### Functional performance

Alves et al. [30] used two hop tests to assess functional performance. They found no significant differences in performance between groups with or without tape in the lateral hop test (P = 0.490) or the figure-of- 8 hop test (P = 0.380). However, there was a significant difference in the figure-of- 8 test after taping (P = 0.026). Baseline scores for both groups were similar, but the Mulligan group showed slightly lower scores after taping compared to the controls.

#### Postural control

Alves et al. [30] measured the yo-yo intermittent recovery test, there was a substantial increase in centre of pressure displacement (both anteroposterior and mediolateral) and area for both Mulligan and placebo tapings (*P* = 0.032).

#### Grading of the quality of evidence

We applied GRADE methodology to evaluate evidence quality across outcomes (Table 4). All outcomes received low-quality ratings except SEBT, which was rated very low. These ratings reflect serious risk of bias and imprecision from small samples with wide confidence intervals. Pain assessment (168 participants) showed significant reduction with MWM (MD = − 0.80) despite moderate heterogeneity (I^2^ = 52%). Weight-bearing lunge test (144 participants) demonstrated improved dorsiflexion (MD = 0.90 cm) with low heterogeneity. Range of motion (164 participants) consistently improved (MD = 0.71 cm), while pressure pain threshold (62 participants) showed no significant difference. Y balance test results (75 participants) indicated meaningful improvement (MD = 4.69 cm), and stiffness perception (92 participants) showed no significant change. SEBT received the lowest rating due to significant heterogeneity (I^2^ = 75%) despite showing improvement.

**Table 4:** GRADE Approach Assessment of the Included Studies

Outcome name	Number of included studies	Design of included studies	Mean difference, 95% CI	Heterogeneity	Number of patients in Mulligan group	Number of patients in control	Risk of bias	Inconsistency	Indirectness	Imprecision	Other considerations	Quality
VAS score for pain	Four studies with 168 patients	RCTs	− 0.80 [− 1.19, − 0.41]	I^2^ = 52%, P = 0.10	84	84	Not serious	Not Serious	Not serious	Serious^b^	Not existed	Moderate ⊕ ⊕ ⊕ ◯
Weight Bearing Lunge test	Four studies with 144 patients	RCTs	0.90 [0.38, 1.43]	I^2^ = 22%, P = 0.28	72	72	Not serious	Not serious	Not serious	Serious^b^	Not existed	Moderate ⊕ ⊕ ⊕ ◯
Range of motion (cm)	Four studies with 164 patients	RCTs	0.71 [0.62, 0.79]	I^2^ = 29%, P = 0.24	82	82	Not serious	Not serious	Not serious	Not Serious	Not existed	High ⊕ ⊕ ⊕ ⊕
Pressure pain threshold	Four studies with 62 patients	RCTs	− 0.26 [− 4.01, 3.49]	I^2^ = 0%, P = 0.66	32	32	Not serious	Not serious	Not serious	Serious	Not existed	Moderate ⊕ ⊕ ⊕ ◯
Y balance test (YBT)	Four studies with 75 patients	RCTs	4.69 [1.67, 7.70]	I^2^ = 16%, P = 0.02	37	38	Not serious	Not serious	Not serious	Serious^b^	Not existed	Moderate ⊕ ⊕ ⊕ ◯
Stiffness perception	Four studies with 92 patients	RCTs	0.10 [− 0.64, 0.85]	I^2^ = 0%, P = 0.52	46	46	Not serious	Not serious	Not serious	Serious^b^	Not existed	Moderate ⊕ ⊕ ⊕ ◯
star excursion balance test (SEBT)	Two studies with 337 patients	RCTs	3.15 [1.44, 4.86]	I^2^ = 75%, P = 0.01	56	57	Not serious	Serious^c^	Not serious	Serious^b^	Not existed	Low ⊕ ⊕ ◯◯

*RCTs* Randomized Control Trials, *CI* Confidence Interval

^a^ Other considerations are publication bias, large effect, dose response, and plausible confounding factors

^b^ As the analysis included small number of patients with wide confidence interval

^c^ Decreased by two points due to significant heterogeinity. High indicates that we are extremely certain that the actual effect is close to the effect estimate. Moderate indicates that the impact estimate has moderate confidence: the actual effect is likely to be close. Low indicates that the confidence about the result is limited and the true effect can be different from our result

## Discussion

This systematic review and meta-analysis found that MWM significantly reduced the visual analogue scale score for pain. Besides, it was associated with a significant increase in the ROM and WBLT. MWM is designed by Brian Mulligan based on his clinical experience to combine physiological movement with accessory mobilization [34]. During the active joint movement, MWM provides a continuous additional joint glide [35]. Tape is placed after the manual application of MWM to help preserve the glide and correct fibular alignment [36]. Several biomechanical and neurophysiological mechanisms can explain the effect of these types of mobilization on joint performance [37, 38]. In terms of balance parameters, the analysis favoured the MWM group over the control group. However, the SEBT did not differ significantly between both groups regarding anterior and posterolateral direction and a significant favorable results with anteromedial direction.

Our quality assessment revealed varying bias levels across studies. YBT and ROM studies generally showed low risk of bias, while VAS pain assessments raised concerns regarding randomization and intervention deviations. ROM measurements faced selection bias issues, and Pressure Pain Threshold demonstrated high bias risk due to measurement concerns. Balance measures (YBT/SEBT) showed reporting inconsistencies and selection bias. Stiffness perception studies had relatively lower risk, though blinding and measurement concerns existed. All but one GRADE assessment classified all evidence as low quality, reflecting these limitations and indicating caution in interpreting results; the remaining GRADE evaluation (i.e. SEBT) was rated as very low certainty of evidence.

Beyond statistical significance, the clinical relevance of our findings warrants careful consideration. For pain reduction, our pooled mean difference of − 0.92 on VAS approaches the established MCID of 1.0–2.0 points [39], suggesting patients would experience meaningful relief. The ROM improvements (SMD = 1.65) substantially exceed Cohen's benchmark for large effects (0.8) [40], indicating clinically significant mobility gains. For WBLT, the improvement (0.90 cm) falls slightly below the reported MCID of ~ 1.3–1.5 cm [41]. Regarding balance measures, the Y Balance Test improvement (MD = 4.69 cm) approaches the ~ 5 cm threshold needed to exceed typical measurement variability [42], suggesting a potentially meaningful enhancement in dynamic stability. The SEBT improvements varied by direction, with the posterolateral reach showing clinical significance when compared to established minimal detectable changes [43]. For pain pressure threshold, our non-significant finding (SMD = − 0.10) falls well below the meaningful change threshold of ~ 0.5 kg/cm^2^ [44], confirming the lack of clinical relevance. The peroneus longus latency reduction (− 12.85 ms) substantially exceeds the small delays (3–5 ms) typically distinguishing stable from unstable ankles [45], suggesting an important enhancement in protective reflexes. Stiffness perception showed no statistically or clinically significant change.

These findings demonstrate that MWM produces clinically meaningful improvements in ROM, and neuromuscular control, with more modest or negligible effects on other parameters. The strongest clinical benefits appear in improved ankle mobility and dynamic balance, which directly relate to functional performance in daily and athletic activities.

A trial by Alves et al. [30] explored the efficacy of fibular repositioning taping on lower limb performance and peroneus longus latency time, finding that it improved latency but did not enhance static postural control in chronic ankle instability. This supports our results, which show that MWM effectively reduces pain and improves functional outcomes. The mechanisms by which MWM enhances neuromuscular control may parallel those of fibular taping, suggesting that MWM can similarly benefit recovery in ankle sprain rehabilitation.

A trial by Marrón-Gómez [46] et al. compared the efficacy of talocrural manipulation and mobilization with movement as two different mobilization techniques in improving the ankle dorsiflexion measured by WBLT in patients with chronic ankle instability [46]. They found That both methods could improve dorsiflexion and their effect might persist for more than two days. The efficacy of the two techniques is comparable to each other and there was no significant difference between them.

De-la-Morena et al. [47] evaluated the impact of Mulligan tape on balance performance utilizing computerised dynamic post-urography through a blinded randomized trial. They found that Mulligan taping did not affect postural and motor control in healthy participants as measured by computerised dynamic post-urography. However, a major limitation of this trial was that the trial was restricted to healthy individuals with no symptoms, therefore, this evidence cannot be applied to symptomatic patients with acute or chronic ankle instability.

Delahunt et al. [48] reported similar results. They found that MWM using repositioning fibular tape did not affect the Star Excursion Balance test. These results were similar to our findings. However, our analysis reported significant improvement in the Y balance test in the MWM group. The Y Balance Test is a commercially available balance measurement instrument that employs three of the eight SEBT orientations (anterior, posteromedial, and posterolateral) and has been suggested as a way of testing dynamic balance [49].

Collins et al. [29] performed a cross-over study double-blinded trial to evaluate the effect of MWM on dorsiflexion and pain perception in patients with subacute ankle subluxation. They found that in subacute ankle sprains, the MWM therapy produced a mechanical rather than a hypoalgesic impact. However, they reported that MWM did not influence the initiation of mechanical movement or thermal pain threshold measurements. MWM method has a direct hypoalgesic effect and mechanical action as it reduces the anterior talus displacement. Excessive anterior talofibular displacement is thought to arise following plantarflexion/inversion injury and persist with anterior talofibular ligament laxity[50]. Therefore, the effect of MWM in reducing the anterior talofibular displacement would improve the dorsiflexion ROM.

According to the literature, the difficulty of sliding the tibia over the talus can restrict dorsiflexion in a closed kinetic chain, limiting knee flexion and decreasing the ability to absorb eccentric loads [51]. Some joint mobilization procedures and strategies are recognized for recovering the dorsiflexion range of movement [29, 52]. However, there is currently no agreement in the research on the addition of clinical effects on ankle dorsiflexion range of movement in the execution of these procedures, particularly when the two best-recognized techniques are combined: the Mulligan Concept and the Maitland technique. In addition, no trials assessing impact maintenance, whether immediate or short-term, were discovered.

This systematic review and meta-analysis provide pooled analysis of all published trials that investigated the efficacy of MWM on patients who had ankle sprains. We included only RCTs with an overall low risk of bias. Besides, the analysis of most outcomes was homogenous. This, in turn, provides high-quality evidence according to GRADE [24].

The quality of evidence significantly influences the interpretation of our findings. Most outcomes were rated as moderate quality, supporting the reliability of the positive effects of MWM on pain reduction and range of motion. However, the SEBT was rated as low quality, indicating a need for further research to confirm its effectiveness. This suggests that while our results are encouraging, additional studies are essential to strengthen the evidence base for MWM's impact on balance outcomes.

In addition, our study has limitations. This study's primary limitation is the potential for bias in the included RCTs, especially concerning randomization and outcome reporting. Although the majority of studies exhibited a low risk of bias in various domains, certain concerns persist, necessitating careful interpretation. Future research must prioritize the enhancement of methodological rigor to improve reliability. Another primary concern is the small number of included studies, which contributes to imprecision and affects the overall quality of evidence. Additionally, there was notable heterogeneity in the analysis of the SEBT. We identified variance in follow-up times among the studies as a key factor influencing this heterogeneity. Another limitation was the inability to include EMBASE in our database search due to institutional access constraints. However, we attempted to minimize this limitation's impact by utilizing Scopus, which has substantial overlap with EMBASE content, along with comprehensive searching of other major databases (Cochrane Central, PubMed, PEDro, and Web of Science), and thorough reference list checking of included studies. Finally, The restriction to English-language studies represents a limitation of this review, as it may have led to language bias and the potential exclusion of relevant studies published in other languages.

## Conclusion

Our study revealed MWM substantially decreased pain levels. Moreover, it was associated with a significant increase in both ROM and WBLT scores. Additionally, the MWM group demonstrated superior performance in balance parameters and posterolateral SEBT compared to the control group. However, both groups showed similar outcomes in regarding pain pressure threshold and stiffness perception. As a result of imprecision and inconsistency, the GRADE evaluation rated the overall findings as being of a low quality.

## Ethics approval and consent to participate

Not applicable.

## Consent for publication

Not applicable.

## Supplementary Information

Supplemantary Material 1.

Supplemantary Material 2.

Supplemantary Material 3.

Supplemantary Material 4.

Supplemantary Material 5.

## Data Availability

The datasets used and/or analysed during the current study are available from the corresponding author on reasonable request.

## Abbreviations

CAI: Chronic ankle instability
GRADE: Grades of Recommendation, Assessment, Development, and Evaluation
MD: Mean difference
SMD: Standardized mean difference
MWM: Mobilization with movement
PRISMA: Preferred Reporting Items for Systematic Reviews and Meta-Analyses
RCTs: Randomized controlled trials
ROM: Range of motion
SEBT: Star excursion balance test
SMD: Standard mean difference
WBLT: Weight-bearing lunge test
