# Supplementary material for: The effectiveness of mulligan mobilization with movement (MWM) on outcomes of patients with ankle sprain: a systematic review and meta-analysis
Source: BMC Sports Sci Med Rehabil. 2025 Apr 29;17:105. doi: 10.1186/s13102-025-01121-6 (PMC12042638; doi:10.1186/s13102-025-01121-6)
Supplement: Supplementary file 1 — Supplemantary Material 1. [file 13102_2025_1121_MOESM1_ESM.docx]

**Supplementary file**

**The Effectiveness of MWM on ankle sprain**

**Search strategies**

PubMed search:

("mobilization with movement"[Title/Abstract] OR "mobilisation with movement"[Title/Abstract] OR "MWM"[Title/Abstract] OR "Mulligan"[Title/Abstract] OR "mulligan's mobilization"[Title/Abstract] OR "mulligan concept"[Title/Abstract] OR "mulligan method"[Title/Abstract] OR "mulligan technique"[Title/Abstract] OR "mobilization with movement*"[Title/Abstract] OR "mobilisation with movement*"[Title/Abstract]) AND ("ankle injuries"[MeSH Terms] OR "ankle injuries"[MeSH Terms] OR "ankle"[Title/Abstract] OR "lateral ligament ankle"[Title/Abstract] OR "deltoid ligament"[Title/Abstract] OR "tibiotalar ligament"[Title/Abstract] OR "tibiocalcaneal ligament"[Title/Abstract] OR "tibionavicular ligament"[Title/Abstract] OR "tibiofibular ligament"[Title/Abstract] OR "ankle sprain"[Title/Abstract] OR "ankle ligament*"[Title/Abstract] OR "syndesmotic injurie*"[Title/Abstract] OR "ankle syndesmosis"[Title/Abstract]) AND ("randomized controlled trial"[Title/Abstract] OR "randomized"[Title/Abstract] OR "randomised"[Title/Abstract] OR "controlled trial"[Title/Abstract] OR "clinical trial"[Title/Abstract] OR "trial"[Title/Abstract] OR "randomisation"[Title/Abstract] OR "randomization"[Title/Abstract] OR "controlled clinical trial"[Title/Abstract] OR "randomly"[Title/Abstract])

Scopus search:

"mobilization with movement" OR "mobilisation with movement" OR MWM OR Mulligan OR "mulligan's mobilization" OR "mulligan concept" OR "mulligan method" OR "mulligan technique" OR "mobilization*-with-movement*" OR "mobilisation*-with-movement*"

ankle injury OR ankle injuries OR ankle OR Lateral Ligament, ankle OR "deltoid ligament" OR "tibiotalar ligament" OR "tibiocalcaneal ligament" OR "tibionavicular ligament" OR "tibiofibular ligament" OR "ankle sprain" OR "ankle ligament*"OR "Syndesmotic Injurie*"OR "ankle syndesmosis"

randomized controlled trial OR randomized OR randomised OR controlled trial OR clinical trial OR trial OR randomisation OR randomization OR controlled clinical trial OR randomly

Web of science search: From 1900 to 2022

Cochrane CENTRAL search:

"mobilization with movement" OR "mobilisation with movement" OR MWM OR Mulligan OR "mulligan's mobilization" OR "mulligan concept" OR "mulligan method" OR "mulligan technique" OR "mobilization*-with-movement*" OR "mobilisation*-with-movement*"

AND

ankle injury OR ankle injuries OR ankle OR Lateral Ligament, ankle OR "deltoid ligament" OR "tibiotalar ligament" OR "tibiocalcaneal ligament" OR "tibionavicular ligament" OR "tibiofibular ligament" OR "ankle sprain" OR "ankle ligament*"OR "Syndesmotic Injurie*"OR "ankle syndesmosis"

AND

randomized controlled trial OR randomized OR randomised OR controlled trial OR clinical trial OR trial OR randomisation OR randomization OR controlled clinical trial OR randomly

PEDRO search:

mwm AND “Ankle sprain”

mulligan AND “ankle sprain”

mwm AND ankle

mulligan AND ankle

Limits: Method: clinical trial
