# Supplementary material for: The effectiveness of mulligan mobilization with movement (MWM) on outcomes of patients with ankle sprain: a systematic review and meta-analysis
Source: BMC Sports Sci Med Rehabil. 2025 Apr 29;17:105. doi: 10.1186/s13102-025-01121-6 (PMC12042638; doi:10.1186/s13102-025-01121-6)
Supplement: Supplementary file 4 — Supplemantary Material 4. [file 13102_2025_1121_MOESM4_ESM.docx]

Supplementary table 1: Risk of bias assessment for each Outcome of interest using the ROB-2 tool of Cochrane.

| Study ID | Outcomes | Randomization Process | Deviations from Intended Interventions | Missing Outcome Data | Measurement of the Outcome | Selection of Reported Result | Overall Risk of Bias |
| --- | --- | --- | --- | --- | --- | --- | --- |
| Gogate 2020 | Ankle ROM | Low | Low | Low | Low | Some concern | Some concern |
|  | Y Balance Test | Low | Low | Low | Low | Some concern | Some concern |
|  | Pain Pressure Threshold | Low | Low | Low | Some concern | Some concern | Some concern |
| Norouzi 2021 | Pain intensity (VAS) | Low | Low | Low | Some concern | Low | Some concern |
|  | Ankle ROM | Low | Low | Low | Low | Low | Low |
| Simsek 2018 | Pain intensity (VAS) | Some concern | Low | Low | Some concern | Some concern | Some concern |
|  | Ankle ROM | Some concern | Low | Low | Low | Some concern | Some concern |
|  | SEBT | Some concern | Low | Low | Low | Some concern | Some concern |
| Nguyen 2020 | Pain intensity (VAS) | Low | Low | Low | Some concern | Some concern | Some concern |
|  | Ankle ROM | Low | Low | Low | Low | Some concern | Some concern |
|  | Stiffness Perception | Low | Low | Low | Some concern | Some concern | Some concern |
| Nguyen 2021 | Pain intensity (VAS) | Some concern | Low | Low | Some concern | Low | Some concern |
|  | Ankle ROM | Some concern | Low | Low | Low | Low | Some concern |
|  | Stiffness Perception | Some concern | Low | Low | Some concern | Low | Some concern |
|  | Y Balance Test | Some concern | Low | Low | Low | Low | Some concern |
| Collins 2004 | Pain Pressure Threshold | Some concern | Low | Low | Some concern | Some concern | Some concern |
|  | Ankle ROM | Some concern | Low | Low | Low | Some concern | Some concern |
| Alves 2018 | Peroneus Longus Latency Time | Some concern | Some concern | Low | Some concern | Some concern | Some concern |
| Cruz-Díaz 2014 | Ankle ROM | Low | Low | Low | Low | Some concern | Some concern |
|  | SEBT | Low | Low | Low | Low | Some concern | Some concern |
| Shadegani 2023 | Peroneus Longus Latency Time | Low | Low | Low | Low | Low | Low |
| Reid 2007 | Ankle ROM | Some concern | Low | Low | Low | Some concern | Some concern |
