# Supplementary material for: The effectiveness of mulligan mobilization with movement (MWM) on outcomes of patients with ankle sprain: a systematic review and meta-analysis
Source: BMC Sports Sci Med Rehabil. 2025 Apr 29;17:105. doi: 10.1186/s13102-025-01121-6 (PMC12042638; doi:10.1186/s13102-025-01121-6)
Supplement: Supplementary file 5 — Supplemantary Material 5. [file 13102_2025_1121_MOESM5_ESM.docx]

| **Unique ID** | 1 | **Study ID** | Gogate 2020 | **Assessor** |  |
| --- | --- | --- | --- | --- | --- |
| **Ref or Label** |  | **Aim** | assignment to intervention (the 'intention-to-treat' effect) |  |  |
| **Experimental** |  | **Comparator** |  | **Source** |  |
| **Outcome** | Ankle ROM | **Results** |  | **Weight** | 1 |
| **Domain** | **Signalling question** | | | **Response** | **Comments** |
| **Bias arising from the randomization process** | 1.1 Was the allocation sequence random? | | | Y |  |
|  | 1.2 Was the allocation sequence concealed until participants were enrolled and assigned to interventions? | | | Y |  |
|  | 1.3 Did baseline differences between intervention groups suggest a problem with the randomization process? | | | N |  |
|  | **Risk of bias judgement** | | | **Low** | The study used a computer-generated randomization sequence prepared by an independent statistician and allocation was concealed in sequentially numbered opaque sealed envelopes |
| **Bias due to deviations from intended interventions** | 2.1.Were participants aware of their assigned intervention during the trial? | | | N |  |
|  | 2.2.Were carers and people delivering the interventions aware of participants' assigned intervention during the trial? | | | N |  |
|  | 2.3. If Y/PY/NI to 2.1 or 2.2: Were there deviations from the intended intervention that arose because of the experimental context? | | | NA |  |
|  | 2.4 If Y/PY to 2.3: Were these deviations likely to have affected the outcome? | | | NA |  |
|  | 2.5. If Y/PY/NI to 2.4: Were these deviations from intended intervention balanced between groups? | | | NA |  |
|  | 2.6 Was an appropriate analysis used to estimate the effect of assignment to intervention? | | | Y |  |
|  | 2.7 If N/PN/NI to 2.6: Was there potential for a substantial impact (on the result) of the failure to analyse participants in the group to which they were randomized? | | | NA |  |
|  | **Risk of bias judgement** | | | **Low** | The study employed blinding of participants and assessors, and participants were instructed not to reveal group identity. The intervention followed a rigorous protocol, ensuring adherence |
| **Bias due to missing outcome data** | 3.1 Were data for this outcome available for all, or nearly all, participants randomized? | | | Y |  |
|  | 3.2 If N/PN/NI to 3.1: Is there evidence that result was not biased by missing outcome data? | | | NA |  |
|  | 3.3 If N/PN to 3.2: Could missingness in the outcome depend on its true value? | | | NA |  |
|  | 3.4 If Y/PY/NI to 3.3: Is it likely that missingness in the outcome depended on its true value? | | | NA |  |
|  | **Risk of bias judgement** | | | **Low** | All participants completed the six treatment sessions, with minimal loss to follow-up, and an intention-to-treat analysis was performed​. |
| **Bias in measurement of the outcome** | 4.1 Was the method of measuring the outcome inappropriate? | | | N | The Foot and Ankle Disability index (FADI) Sports module was used as a measure of functional limitation associated with foot and ankle injury. |
|  | 4.2 Could measurement or ascertainment of the outcome have differed between intervention groups? | | | N |  |
|  | 4.3 Were outcome assessors aware of the intervention received by study participants? | | | N |  |
|  | 4.4 If Y/PY/NI to 4.3: Could assessment of the outcome have been influenced by knowledge of intervention received? | | | NA |  |
|  | 4.5 If Y/PY/NI to 4.4: Is it likely that assessment of the outcome was influenced by knowledge of intervention received? | | | NA |  |
|  | **Risk of bias judgement** | | | **Low** | A blinded physiotherapist assessed outcomes at baseline, post-treatment, 1 month, and 6 months. Validated tools (NRS, FADI) were used. |
| **Bias in selection of the reported result** | 5.1 Were the data that produced this result analysed in accordance with a pre-specified analysis plan that was finalized before unblinded outcome data were available for analysis? | | | N |  |
|  | 5.2 ... multiple eligible outcome measurements (e.g. scales, definitions, time points) within the outcome domain? | | | N |  |
|  | 5.3 ... multiple eligible analyses of the data? | | | N |  |
|  | **Risk of bias judgement** | | | **Some concerns** | The specific registration number is not available. |
| **Overall bias** | **Risk of bias judgement** | | | **Some concerns** |  |
|  |  |  |  |  |  |
|  |  |  |  |  |  |
| **Unique ID** | 2 | **Study ID** | Norouzi 2021 | **Assessor** |  |
| **Ref or Label** |  | **Aim** | assignment to intervention (the 'intention-to-treat' effect) |  |  |
| **Experimental** |  | **Comparator** |  | **Source** |  |
| **Outcome** | Ankle ROM | **Results** |  | **Weight** | 1 |
| **Domain** | **Signalling question** | | | **Response** | **Comments** |
| **Bias arising from the randomization process** | 1.1 Was the allocation sequence random? | | | Y |  |
|  | 1.2 Was the allocation sequence concealed until participants were enrolled and assigned to interventions? | | | Y |  |
|  | 1.3 Did baseline differences between intervention groups suggest a problem with the randomization process? | | | N |  |
|  | **Risk of bias judgement** | | | **Low** | The study mentions that participants were randomly assigned to one of the two groups using sealed envelopes containing numbers from 1 to 40. The use of sealed envelopes for allocation is a good method to ensure allocation concealment, which reduces the risk of selection bias. |
| **Bias due to deviations from intended interventions** | 2.1.Were participants aware of their assigned intervention during the trial? | | | N |  |
|  | 2.2.Were carers and people delivering the interventions aware of participants' assigned intervention during the trial? | | | Y |  |
|  | 2.3. If Y/PY/NI to 2.1 or 2.2: Were there deviations from the intended intervention that arose because of the experimental context? | | | N |  |
|  | 2.4 If Y/PY to 2.3: Were these deviations likely to have affected the outcome? | | | NA |  |
|  | 2.5. If Y/PY/NI to 2.4: Were these deviations from intended intervention balanced between groups? | | | NA |  |
|  | 2.6 Was an appropriate analysis used to estimate the effect of assignment to intervention? | | | Y |  |
|  | 2.7 If N/PN/NI to 2.6: Was there potential for a substantial impact (on the result) of the failure to analyse participants in the group to which they were randomized? | | | NA |  |
|  | **Risk of bias judgement** | | | **Low** | The study was described as double-blind, meaning neither the participants nor the evaluator were aware of the intervention type. However, the therapist delivering the intervention was not blinded, which could introduce performance bias. |
| **Bias due to missing outcome data** | 3.1 Were data for this outcome available for all, or nearly all, participants randomized? | | | PY |  |
|  | 3.2 If N/PN/NI to 3.1: Is there evidence that result was not biased by missing outcome data? | | | NA |  |
|  | 3.3 If N/PN to 3.2: Could missingness in the outcome depend on its true value? | | | NA |  |
|  | 3.4 If Y/PY/NI to 3.3: Is it likely that missingness in the outcome depended on its true value? | | | NA |  |
|  | **Risk of bias judgement** | | | **Low** | The study had a relatively low dropout rate. Out of 40 participants, 4 were excluded before grouping, and 6 dropped out during the intervention (3 from the Maitland group and 3 from the Mulligan group). The final analysis included 33 participants, with no missing outcome data reported.The study used an intention-to-treat analysis, which further mitigates the risk of bias. |
| **Bias in measurement of the outcome** | 4.1 Was the method of measuring the outcome inappropriate? | | | N |  |
|  | 4.2 Could measurement or ascertainment of the outcome have differed between intervention groups? | | | N |  |
|  | 4.3 Were outcome assessors aware of the intervention received by study participants? | | | N |  |
|  | 4.4 If Y/PY/NI to 4.3: Could assessment of the outcome have been influenced by knowledge of intervention received? | | | NA |  |
|  | 4.5 If Y/PY/NI to 4.4: Is it likely that assessment of the outcome was influenced by knowledge of intervention received? | | | NA |  |
|  | **Risk of bias judgement** | | | **Low** | The outcome assessor was blinded to the treatment allocation, which reduces the risk of detection bias.  The primary outcomes (range of motion) were measured using validated tools (Weight Bearing Lunge Test for range of motion).  The WBLT is a well-established, objective measure that minimizes subjective bias by providing quantifiable, reproducible results. |
| **Bias in selection of the reported result** | 5.1 Were the data that produced this result analysed in accordance with a pre-specified analysis plan that was finalized before unblinded outcome data were available for analysis? | | | Y |  |
|  | 5.2 ... multiple eligible outcome measurements (e.g. scales, definitions, time points) within the outcome domain? | | | N |  |
|  | 5.3 ... multiple eligible analyses of the data? | | | N |  |
|  | **Risk of bias judgement** | | | **Low** | The study protocol was registered prospectively (IRCT20190108042292N2), and the outcomes were pre-specified. This reduces the risk of selective reporting. |
| **Overall bias** | **Risk of bias judgement** | | | **Low** |  |
|  |  |  |  |  |  |
|  |  |  |  |  |  |
| **Unique ID** | 3 | **Study ID** | Simsek 2018 | **Assessor** |  |
| **Ref or Label** |  | **Aim** | assignment to intervention (the 'intention-to-treat' effect) |  |  |
| **Experimental** |  | **Comparator** |  | **Source** |  |
| **Outcome** | Pain intensity (VAS) | **Results** |  | **Weight** | 1 |
| **Domain** | **Signalling question** | | | **Response** | **Comments** |
| **Bias arising from the randomization process** | 1.1 Was the allocation sequence random? | | | NI |  |
|  | 1.2 Was the allocation sequence concealed until participants were enrolled and assigned to interventions? | | | NI |  |
|  | 1.3 Did baseline differences between intervention groups suggest a problem with the randomization process? | | | N |  |
|  | **Risk of bias judgement** | | | **Some concerns** | The study states that randomization was performed but does not provide details about the method of random sequence generation or allocation concealment. This lack of transparency introduces potential selection bias. |
| **Bias due to deviations from intended interventions** | 2.1.Were participants aware of their assigned intervention during the trial? | | | PY |  |
|  | 2.2.Were carers and people delivering the interventions aware of participants' assigned intervention during the trial? | | | PY |  |
|  | 2.3. If Y/PY/NI to 2.1 or 2.2: Were there deviations from the intended intervention that arose because of the experimental context? | | | N |  |
|  | 2.4 If Y/PY to 2.3: Were these deviations likely to have affected the outcome? | | | NA |  |
|  | 2.5. If Y/PY/NI to 2.4: Were these deviations from intended intervention balanced between groups? | | | NA |  |
|  | 2.6 Was an appropriate analysis used to estimate the effect of assignment to intervention? | | | Y |  |
|  | 2.7 If N/PN/NI to 2.6: Was there potential for a substantial impact (on the result) of the failure to analyse participants in the group to which they were randomized? | | | NA |  |
|  | **Risk of bias judgement** | | | **Low** | The study followed a clear protocol for intervention application (fibular taping technique), and participants were assessed using standardized outcome measures. There is no indication that deviations occurred. In addition, the study does not mention whether participants or therapists were blinded to the intervention. Given that the intervention involves taping, it is unlikely that participants were blinded, which could introduce performance bias. |
| **Bias due to missing outcome data** | 3.1 Were data for this outcome available for all, or nearly all, participants randomized? | | | Y |  |
|  | 3.2 If N/PN/NI to 3.1: Is there evidence that result was not biased by missing outcome data? | | | NA |  |
|  | 3.3 If N/PN to 3.2: Could missingness in the outcome depend on its true value? | | | NA |  |
|  | 3.4 If Y/PY/NI to 3.3: Is it likely that missingness in the outcome depended on its true value? | | | NA |  |
|  | **Risk of bias judgement** | | | **Low** | No major loss to follow-up was reported. The results include data from all 26 participants, and no selective exclusion of data was noted. |
| **Bias in measurement of the outcome** | 4.1 Was the method of measuring the outcome inappropriate? | | | N |  |
|  | 4.2 Could measurement or ascertainment of the outcome have differed between intervention groups? | | | N |  |
|  | 4.3 Were outcome assessors aware of the intervention received by study participants? | | | Y |  |
|  | 4.4 If Y/PY/NI to 4.3: Could assessment of the outcome have been influenced by knowledge of intervention received? | | | PY |  |
|  | 4.5 If Y/PY/NI to 4.4: Is it likely that assessment of the outcome was influenced by knowledge of intervention received? | | | PN |  |
|  | **Risk of bias judgement** | | | **Some concerns** | The primary outcomes (pain) were measured using validated tools (VAS). However, pain is a subjective measure, and the lack of blinding of the assessor could influence the results. |
| **Bias in selection of the reported result** | 5.1 Were the data that produced this result analysed in accordance with a pre-specified analysis plan that was finalized before unblinded outcome data were available for analysis? | | | NI |  |
|  | 5.2 ... multiple eligible outcome measurements (e.g. scales, definitions, time points) within the outcome domain? | | | N |  |
|  | 5.3 ... multiple eligible analyses of the data? | | | N |  |
|  | **Risk of bias judgement** | | | **Some concerns** | While the study presents results for all planned outcomes, no trial registration or protocol reference was found, making it unclear whether all pre-specified outcomes were reported. |
| **Overall bias** | **Risk of bias judgement** | | | **Some concerns** |  |
|  |  |  |  |  |  |
|  |  |  |  |  |  |
| **Unique ID** | 4 | **Study ID** | Nguyen 2020 | **Assessor** |  |
| **Ref or Label** |  | **Aim** | assignment to intervention (the 'intention-to-treat' effect) |  |  |
| **Experimental** |  | **Comparator** |  | **Source** |  |
| **Outcome** | Ankle ROM | **Results** |  | **Weight** | 1 |
| **Domain** | **Signalling question** | | | **Response** | **Comments** |
| **Bias arising from the randomization process** | 1.1 Was the allocation sequence random? | | | Y |  |
|  | 1.2 Was the allocation sequence concealed until participants were enrolled and assigned to interventions? | | | Y |  |
|  | 1.3 Did baseline differences between intervention groups suggest a problem with the randomization process? | | | N |  |
|  | **Risk of bias judgement** | | | **Low** | The study used computer-generated randomization, and allocation concealment was maintained using opaque sealed envelopes |
| **Bias due to deviations from intended interventions** | 2.1.Were participants aware of their assigned intervention during the trial? | | | N |  |
|  | 2.2.Were carers and people delivering the interventions aware of participants' assigned intervention during the trial? | | | Y |  |
|  | 2.3. If Y/PY/NI to 2.1 or 2.2: Were there deviations from the intended intervention that arose because of the experimental context? | | | N |  |
|  | 2.4 If Y/PY to 2.3: Were these deviations likely to have affected the outcome? | | | NA |  |
|  | 2.5. If Y/PY/NI to 2.4: Were these deviations from intended intervention balanced between groups? | | | NA |  |
|  | 2.6 Was an appropriate analysis used to estimate the effect of assignment to intervention? | | | Y |  |
|  | 2.7 If N/PN/NI to 2.6: Was there potential for a substantial impact (on the result) of the failure to analyse participants in the group to which they were randomized? | | | NA |  |
|  | **Risk of bias judgement** | | | **Low** | Participants and healthcare providers were blinded to the assigned interventions. There were no reported protocol deviations affecting the primary outcome. |
| **Bias due to missing outcome data** | 3.1 Were data for this outcome available for all, or nearly all, participants randomized? | | | Y |  |
|  | 3.2 If N/PN/NI to 3.1: Is there evidence that result was not biased by missing outcome data? | | | NA |  |
|  | 3.3 If N/PN to 3.2: Could missingness in the outcome depend on its true value? | | | NA |  |
|  | 3.4 If Y/PY/NI to 3.3: Is it likely that missingness in the outcome depended on its true value? | | | NA |  |
|  | **Risk of bias judgement** | | | **Low** | All randomized participants were included in the intention-to-treat analysis. Dropout rates were minimal, and missing data were handled appropriately. |
| **Bias in measurement of the outcome** | 4.1 Was the method of measuring the outcome inappropriate? | | | N |  |
|  | 4.2 Could measurement or ascertainment of the outcome have differed between intervention groups? | | | N |  |
|  | 4.3 Were outcome assessors aware of the intervention received by study participants? | | | N |  |
|  | 4.4 If Y/PY/NI to 4.3: Could assessment of the outcome have been influenced by knowledge of intervention received? | | | NA |  |
|  | 4.5 If Y/PY/NI to 4.4: Is it likely that assessment of the outcome was influenced by knowledge of intervention received? | | | NA |  |
|  | **Risk of bias judgement** | | | **Low** | Outcome assessors were blinded, and validated measurement tools were used to collect data. The ankle dorsiflexion range of motion (ROM) was measured using the Weight Bearing Lunge (WBL) test, which is an objective, tool-based assessment rather than a subjective patient-reported measure. |
| **Bias in selection of the reported result** | 5.1 Were the data that produced this result analysed in accordance with a pre-specified analysis plan that was finalized before unblinded outcome data were available for analysis? | | | NI |  |
|  | 5.2 ... multiple eligible outcome measurements (e.g. scales, definitions, time points) within the outcome domain? | | | N |  |
|  | 5.3 ... multiple eligible analyses of the data? | | | N |  |
|  | **Risk of bias judgement** | | | **Some concerns** | All planned outcomes were reported without evidence of selective reporting. However, no trial registration number found in a recognized clinical trial registry. |
| **Overall bias** | **Risk of bias judgement** | | | **Some concerns** |  |
|  |  |  |  |  |  |
|  |  |  |  |  |  |
| **Unique ID** | 5 | **Study ID** | Nguyen 2021 | **Assessor** |  |
| **Ref or Label** |  | **Aim** | assignment to intervention (the 'intention-to-treat' effect) |  |  |
| **Experimental** |  | **Comparator** |  | **Source** |  |
| **Outcome** | Ankle ROM | **Results** |  | **Weight** | 1 |
| **Domain** | **Signalling question** | | | **Response** | **Comments** |
| **Bias arising from the randomization process** | 1.1 Was the allocation sequence random? | | | Y |  |
|  | 1.2 Was the allocation sequence concealed until participants were enrolled and assigned to interventions? | | | NI |  |
|  | 1.3 Did baseline differences between intervention groups suggest a problem with the randomization process? | | | N |  |
|  | **Risk of bias judgement** | | | **Some concerns** | The study used an independent operator to randomize participants into two groups (MWM or Sham) using a randomized sequence generated via Excel 2010. |
| **Bias due to deviations from intended interventions** | 2.1.Were participants aware of their assigned intervention during the trial? | | | N |  |
|  | 2.2.Were carers and people delivering the interventions aware of participants' assigned intervention during the trial? | | | Y |  |
|  | 2.3. If Y/PY/NI to 2.1 or 2.2: Were there deviations from the intended intervention that arose because of the experimental context? | | | N |  |
|  | 2.4 If Y/PY to 2.3: Were these deviations likely to have affected the outcome? | | | NA |  |
|  | 2.5. If Y/PY/NI to 2.4: Were these deviations from intended intervention balanced between groups? | | | NA |  |
|  | 2.6 Was an appropriate analysis used to estimate the effect of assignment to intervention? | | | Y |  |
|  | 2.7 If N/PN/NI to 2.6: Was there potential for a substantial impact (on the result) of the failure to analyse participants in the group to which they were randomized? | | | NA |  |
|  | **Risk of bias judgement** | | | **Low** | Participants were kept blinded to their group allocation throughout the experiment​ A non-blinded physiotherapist applied the interventions, but a blinded postgraduate physiotherapist supervised all outcome measurements. |
| **Bias due to missing outcome data** | 3.1 Were data for this outcome available for all, or nearly all, participants randomized? | | | Y |  |
|  | 3.2 If N/PN/NI to 3.1: Is there evidence that result was not biased by missing outcome data? | | | NA |  |
|  | 3.3 If N/PN to 3.2: Could missingness in the outcome depend on its true value? | | | NA |  |
|  | 3.4 If Y/PY/NI to 3.3: Is it likely that missingness in the outcome depended on its true value? | | | NA |  |
|  | **Risk of bias judgement** | | | **Low** | No participants were lost to follow-up in either group. |
| **Bias in measurement of the outcome** | 4.1 Was the method of measuring the outcome inappropriate? | | | N |  |
|  | 4.2 Could measurement or ascertainment of the outcome have differed between intervention groups? | | | N |  |
|  | 4.3 Were outcome assessors aware of the intervention received by study participants? | | | N |  |
|  | 4.4 If Y/PY/NI to 4.3: Could assessment of the outcome have been influenced by knowledge of intervention received? | | | NA |  |
|  | 4.5 If Y/PY/NI to 4.4: Is it likely that assessment of the outcome was influenced by knowledge of intervention received? | | | NA |  |
|  | **Risk of bias judgement** | | | **Low** | The same blinded physiotherapist assessed all outcomes​. Measures such as dorsiflexion ROM, pain scales, and balance tests were used, reducing the risk of bias. The ankle dorsiflexion range of motion (ROM) was measured using the Weight Bearing Lunge (WBL) test, which is an objective, tool-based assessment rather than a subjective patient-reported measure. |
| **Bias in selection of the reported result** | 5.1 Were the data that produced this result analysed in accordance with a pre-specified analysis plan that was finalized before unblinded outcome data were available for analysis? | | | Y |  |
|  | 5.2 ... multiple eligible outcome measurements (e.g. scales, definitions, time points) within the outcome domain? | | | N |  |
|  | 5.3 ... multiple eligible analyses of the data? | | | N |  |
|  | **Risk of bias judgement** | | | **Low** | The study was registered on ClinicalTrials.gov (NCT03948503). In addition, No evidence suggests selective reporting of outcomes. |
| **Overall bias** | **Risk of bias judgement** | | | **Some concerns** |  |
|  |  |  |  |  |  |
|  |  |  |  |  |  |
| **Unique ID** | 6 | **Study ID** | Collins 2004 | **Assessor** |  |
| **Ref or Label** |  | **Aim** | assignment to intervention (the 'intention-to-treat' effect) |  |  |
| **Experimental** |  | **Comparator** |  | **Source** |  |
| **Outcome** | Ankle ROM | **Results** |  | **Weight** | 1 |
| **Domain** | **Signalling question** | | | **Response** | **Comments** |
| **Bias arising from the randomization process** | 1.1 Was the allocation sequence random? | | | NI |  |
|  | 1.2 Was the allocation sequence concealed until participants were enrolled and assigned to interventions? | | | NI |  |
|  | 1.3 Did baseline differences between intervention groups suggest a problem with the randomization process? | | | N |  |
|  | **Risk of bias judgement** | | | **Some concerns** | The study states that it was a double-blind randomized controlled trial, but it does not provide details on the method of random sequence generation or allocation concealment. Without this information, the potential for selection bias cannot be ruled out. |
| **Bias due to deviations from intended interventions** | 2.1.Were participants aware of their assigned intervention during the trial? | | | N |  |
|  | 2.2.Were carers and people delivering the interventions aware of participants' assigned intervention during the trial? | | | N |  |
|  | 2.3. If Y/PY/NI to 2.1 or 2.2: Were there deviations from the intended intervention that arose because of the experimental context? | | | NA |  |
|  | 2.4 If Y/PY to 2.3: Were these deviations likely to have affected the outcome? | | | NA |  |
|  | 2.5. If Y/PY/NI to 2.4: Were these deviations from intended intervention balanced between groups? | | | NA |  |
|  | 2.6 Was an appropriate analysis used to estimate the effect of assignment to intervention? | | | Y |  |
|  | 2.7 If N/PN/NI to 2.6: Was there potential for a substantial impact (on the result) of the failure to analyse participants in the group to which they were randomized? | | | NA |  |
|  | **Risk of bias judgement** | | | **Low** | The intervention (Mulligan’s mobilization with movement) and placebo conditions were properly administered, and patients in both groups followed the intended treatments. There were no reported deviations from the assigned interventions. |
| **Bias due to missing outcome data** | 3.1 Were data for this outcome available for all, or nearly all, participants randomized? | | | Y |  |
|  | 3.2 If N/PN/NI to 3.1: Is there evidence that result was not biased by missing outcome data? | | | NA |  |
|  | 3.3 If N/PN to 3.2: Could missingness in the outcome depend on its true value? | | | NA |  |
|  | 3.4 If Y/PY/NI to 3.3: Is it likely that missingness in the outcome depended on its true value? | | | NA |  |
|  | **Risk of bias judgement** | | | **Low** | The study did not report missing data that could affect the outcome. No participants were lost to follow-up, and the results include data from all enrolled participants. |
| **Bias in measurement of the outcome** | 4.1 Was the method of measuring the outcome inappropriate? | | | N |  |
|  | 4.2 Could measurement or ascertainment of the outcome have differed between intervention groups? | | | N |  |
|  | 4.3 Were outcome assessors aware of the intervention received by study participants? | | | N |  |
|  | 4.4 If Y/PY/NI to 4.3: Could assessment of the outcome have been influenced by knowledge of intervention received? | | | NA |  |
|  | 4.5 If Y/PY/NI to 4.4: Is it likely that assessment of the outcome was influenced by knowledge of intervention received? | | | NA |  |
|  | **Risk of bias judgement** | | | **Low** | The WBL test provides an objective, standardized, and reliable method for assessing ankle dorsiflexion ROM. Since it is based on a measurable distance rather than subjective patient feedback, it minimizes bias and ensures accurate results. |
| **Bias in selection of the reported result** | 5.1 Were the data that produced this result analysed in accordance with a pre-specified analysis plan that was finalized before unblinded outcome data were available for analysis? | | | NI |  |
|  | 5.2 ... multiple eligible outcome measurements (e.g. scales, definitions, time points) within the outcome domain? | | | N |  |
|  | 5.3 ... multiple eligible analyses of the data? | | | N |  |
|  | **Risk of bias judgement** | | | **Some concerns** | There is no mention of a trial registration or pre-specified protocol. This makes it unclear whether all outcomes were reported as planned or if selective reporting occurred. |
| **Overall bias** | **Risk of bias judgement** | | | **Some concerns** |  |
|  |  |  |  |  |  |
|  |  |  |  |  |  |
| **Unique ID** | 7 | **Study ID** | Alves 2018 | **Assessor** |  |
| **Ref or Label** |  | **Aim** | assignment to intervention (the 'intention-to-treat' effect) |  |  |
| **Experimental** |  | **Comparator** |  | **Source** |  |
| **Outcome** | Peroneus Longus Latency Time | **Results** |  | **Weight** | 1 |
| **Domain** | **Signalling question** | | | **Response** | **Comments** |
| **Bias arising from the randomization process** | 1.1 Was the allocation sequence random? | | | Y |  |
|  | 1.2 Was the allocation sequence concealed until participants were enrolled and assigned to interventions? | | | NI |  |
|  | 1.3 Did baseline differences between intervention groups suggest a problem with the randomization process? | | | N |  |
|  | **Risk of bias judgement** | | | **Some concerns** | The study used block randomization to assign participants to either the Mulligan taping or placebo taping condition. The randomization was performed using software (Randomizer.org). |
| **Bias due to deviations from intended interventions** | 2.1.Were participants aware of their assigned intervention during the trial? | | | N |  |
|  | 2.2.Were carers and people delivering the interventions aware of participants' assigned intervention during the trial? | | | Y |  |
|  | 2.3. If Y/PY/NI to 2.1 or 2.2: Were there deviations from the intended intervention that arose because of the experimental context? | | | PY |  |
|  | 2.4 If Y/PY to 2.3: Were these deviations likely to have affected the outcome? | | | PY |  |
|  | 2.5. If Y/PY/NI to 2.4: Were these deviations from intended intervention balanced between groups? | | | Y |  |
|  | 2.6 Was an appropriate analysis used to estimate the effect of assignment to intervention? | | | Y |  |
|  | 2.7 If N/PN/NI to 2.6: Was there potential for a substantial impact (on the result) of the failure to analyse participants in the group to which they were randomized? | | | NA |  |
|  | **Risk of bias judgement** | | | **Some concerns** | The study was a crossover trial, and participants received both interventions (Mulligan taping and placebo taping) in a randomized order. The investigators and participants were blinded to the intervention type for postural control and latency time measurements, but blinding was not possible for functional performance tests.   While blinding was maintained for some outcomes, the lack of blinding for functional performance tests could introduce bias. Additionally, the placebo taping was applied without tension, which might not fully mimic the Mulligan taping, potentially affecting the results. |
| **Bias due to missing outcome data** | 3.1 Were data for this outcome available for all, or nearly all, participants randomized? | | | Y |  |
|  | 3.2 If N/PN/NI to 3.1: Is there evidence that result was not biased by missing outcome data? | | | NA |  |
|  | 3.3 If N/PN to 3.2: Could missingness in the outcome depend on its true value? | | | NA |  |
|  | 3.4 If Y/PY/NI to 3.3: Is it likely that missingness in the outcome depended on its true value? | | | NA |  |
|  | **Risk of bias judgement** | | | **Low** | The study reported no significant missing data, and all participants completed the study. However, one participant did not complete the 15-second unipedal stance test, which was excluded from the analysis for that specific outcome. |
| **Bias in measurement of the outcome** | 4.1 Was the method of measuring the outcome inappropriate? | | | N |  |
|  | 4.2 Could measurement or ascertainment of the outcome have differed between intervention groups? | | | N |  |
|  | 4.3 Were outcome assessors aware of the intervention received by study participants? | | | Y |  |
|  | 4.4 If Y/PY/NI to 4.3: Could assessment of the outcome have been influenced by knowledge of intervention received? | | | PY |  |
|  | 4.5 If Y/PY/NI to 4.4: Is it likely that assessment of the outcome was influenced by knowledge of intervention received? | | | N |  |
|  | **Risk of bias judgement** | | | **Some concerns** | The assessor for peroneus longus latency time was blinded, but the functional performance assessor was not, leading to possible measurement bias. |
| **Bias in selection of the reported result** | 5.1 Were the data that produced this result analysed in accordance with a pre-specified analysis plan that was finalized before unblinded outcome data were available for analysis? | | | NI |  |
|  | 5.2 ... multiple eligible outcome measurements (e.g. scales, definitions, time points) within the outcome domain? | | | N |  |
|  | 5.3 ... multiple eligible analyses of the data? | | | N |  |
|  | **Risk of bias judgement** | | | **Some concerns** | The study protocol was not explicitly mentioned, but the outcomes reported align with the methods described. The statistical analysis plan was appropriate, and no selective reporting was evident. |
| **Overall bias** | **Risk of bias judgement** | | | **Some concerns** |  |
|  |  |  |  |  |  |
|  |  |  |  |  |  |
| **Unique ID** | 8 | **Study ID** | Cruz-Diaz 2014 | **Assessor** |  |
| **Ref or Label** |  | **Aim** | assignment to intervention (the 'intention-to-treat' effect) |  |  |
| **Experimental** |  | **Comparator** |  | **Source** |  |
| **Outcome** | Ankle ROM | **Results** |  | **Weight** | 1 |
| **Domain** | **Signalling question** | | | **Response** | **Comments** |
| **Bias arising from the randomization process** | 1.1 Was the allocation sequence random? | | | Y |  |
|  | 1.2 Was the allocation sequence concealed until participants were enrolled and assigned to interventions? | | | Y |  |
|  | 1.3 Did baseline differences between intervention groups suggest a problem with the randomization process? | | | N |  |
|  | **Risk of bias judgement** | | | **Low** | The study used computer-generated randomization to allocate participants to intervention, placebo, or control groups. Allocation was concealed using sealed opaque envelopes and was conducted by an independent administrator who was not involved in eligibility assessment or treatment |
| **Bias due to deviations from intended interventions** | 2.1.Were participants aware of their assigned intervention during the trial? | | | N |  |
|  | 2.2.Were carers and people delivering the interventions aware of participants' assigned intervention during the trial? | | | N |  |
|  | 2.3. If Y/PY/NI to 2.1 or 2.2: Were there deviations from the intended intervention that arose because of the experimental context? | | | NA |  |
|  | 2.4 If Y/PY to 2.3: Were these deviations likely to have affected the outcome? | | | NA |  |
|  | 2.5. If Y/PY/NI to 2.4: Were these deviations from intended intervention balanced between groups? | | | NA |  |
|  | 2.6 Was an appropriate analysis used to estimate the effect of assignment to intervention? | | | Y |  |
|  | 2.7 If N/PN/NI to 2.6: Was there potential for a substantial impact (on the result) of the failure to analyse participants in the group to which they were randomized? | | | NA |  |
|  | **Risk of bias judgement** | | | **Low** | Both participants and investigators were blinded to group allocation. Patients and therapists were instructed not to discuss treatment, and all groups received similar levels of attention. No deviations from protocol were reported that would affect the validity of the results​. |
| **Bias due to missing outcome data** | 3.1 Were data for this outcome available for all, or nearly all, participants randomized? | | | Y |  |
|  | 3.2 If N/PN/NI to 3.1: Is there evidence that result was not biased by missing outcome data? | | | NA |  |
|  | 3.3 If N/PN to 3.2: Could missingness in the outcome depend on its true value? | | | NA |  |
|  | 3.4 If Y/PY/NI to 3.3: Is it likely that missingness in the outcome depended on its true value? | | | NA |  |
|  | **Risk of bias judgement** | | | **Low** | The study reported intent-to-treat (ITT) analysis and used expectation-maximization (EM) imputation for missing values. |
| **Bias in measurement of the outcome** | 4.1 Was the method of measuring the outcome inappropriate? | | | N |  |
|  | 4.2 Could measurement or ascertainment of the outcome have differed between intervention groups? | | | N |  |
|  | 4.3 Were outcome assessors aware of the intervention received by study participants? | | | N |  |
|  | 4.4 If Y/PY/NI to 4.3: Could assessment of the outcome have been influenced by knowledge of intervention received? | | | NA |  |
|  | 4.5 If Y/PY/NI to 4.4: Is it likely that assessment of the outcome was influenced by knowledge of intervention received? | | | NA |  |
|  | **Risk of bias judgement** | | | **Low** | The WBL test provides an objective, standardized, and reliable method for assessing ankle dorsiflexion ROM. Since it is based on a measurable distance rather than subjective patient feedback, it minimizes bias . |
| **Bias in selection of the reported result** | 5.1 Were the data that produced this result analysed in accordance with a pre-specified analysis plan that was finalized before unblinded outcome data were available for analysis? | | | NI |  |
|  | 5.2 ... multiple eligible outcome measurements (e.g. scales, definitions, time points) within the outcome domain? | | | N |  |
|  | 5.3 ... multiple eligible analyses of the data? | | | N |  |
|  | **Risk of bias judgement** | | | **Some concerns** | While the study followed a pre-specified statistical plan, it is unclear if all planned outcomes were reported in full. There is no mention of a pre-registered protocol, which makes it difficult to confirm if selective outcome reporting was avoided​. |
| **Overall bias** | **Risk of bias judgement** | | | **Some concerns** |  |
|  |  |  |  |  |  |
|  |  |  |  |  |  |
| **Unique ID** | 9 | **Study ID** | Shadegani 2023 | **Assessor** |  |
| **Ref or Label** |  | **Aim** | assignment to intervention (the 'intention-to-treat' effect) |  |  |
| **Experimental** |  | **Comparator** |  | **Source** |  |
| **Outcome** | Peroneus Longus Latency Time | **Results** |  | **Weight** | 1 |
| **Domain** | **Signalling question** | | | **Response** | **Comments** |
| **Bias arising from the randomization process** | 1.1 Was the allocation sequence random? | | | Y |  |
|  | 1.2 Was the allocation sequence concealed until participants were enrolled and assigned to interventions? | | | Y |  |
|  | 1.3 Did baseline differences between intervention groups suggest a problem with the randomization process? | | | N |  |
|  | **Risk of bias judgement** | | | **Low** | The study used a web-based randomization system for random sequence generation, ensuring that allocation was not predictable. |
| **Bias due to deviations from intended interventions** | 2.1.Were participants aware of their assigned intervention during the trial? | | | Y |  |
|  | 2.2.Were carers and people delivering the interventions aware of participants' assigned intervention during the trial? | | | N |  |
|  | 2.3. If Y/PY/NI to 2.1 or 2.2: Were there deviations from the intended intervention that arose because of the experimental context? | | | N |  |
|  | 2.4 If Y/PY to 2.3: Were these deviations likely to have affected the outcome? | | | NA |  |
|  | 2.5. If Y/PY/NI to 2.4: Were these deviations from intended intervention balanced between groups? | | | NA |  |
|  | 2.6 Was an appropriate analysis used to estimate the effect of assignment to intervention? | | | Y |  |
|  | 2.7 If N/PN/NI to 2.6: Was there potential for a substantial impact (on the result) of the failure to analyse participants in the group to which they were randomized? | | | NA |  |
|  | **Risk of bias judgement** | | | **Low** | Blinding of participants was not implemented, which may have influenced their behavior and engagement with the intervention. However, outcome assessors were blinded, reducing performance bias. |
| **Bias due to missing outcome data** | 3.1 Were data for this outcome available for all, or nearly all, participants randomized? | | | Y |  |
|  | 3.2 If N/PN/NI to 3.1: Is there evidence that result was not biased by missing outcome data? | | | NA |  |
|  | 3.3 If N/PN to 3.2: Could missingness in the outcome depend on its true value? | | | NA |  |
|  | 3.4 If Y/PY/NI to 3.3: Is it likely that missingness in the outcome depended on its true value? | | | NA |  |
|  | **Risk of bias judgement** | | | **Low** | An intention-to-treat (ITT) analysis was performed, which helps reduce bias from participant dropout​. |
| **Bias in measurement of the outcome** | 4.1 Was the method of measuring the outcome inappropriate? | | | N | objective EMG-based measurements were used, reducing measurement bias. |
|  | 4.2 Could measurement or ascertainment of the outcome have differed between intervention groups? | | | N |  |
|  | 4.3 Were outcome assessors aware of the intervention received by study participants? | | | N |  |
|  | 4.4 If Y/PY/NI to 4.3: Could assessment of the outcome have been influenced by knowledge of intervention received? | | | NA |  |
|  | 4.5 If Y/PY/NI to 4.4: Is it likely that assessment of the outcome was influenced by knowledge of intervention received? | | | NA |  |
|  | **Risk of bias judgement** | | | **Low** | Outcome assessors were blinded, reducing the likelihood of measurement bias.The study used validated measurement tools, minimizing subjective influence.The study used electromyography (EMG) and force platform data, which are less prone to subjective bias. |
| **Bias in selection of the reported result** | 5.1 Were the data that produced this result analysed in accordance with a pre-specified analysis plan that was finalized before unblinded outcome data were available for analysis? | | | Y |  |
|  | 5.2 ... multiple eligible outcome measurements (e.g. scales, definitions, time points) within the outcome domain? | | | N |  |
|  | 5.3 ... multiple eligible analyses of the data? | | | N |  |
|  | **Risk of bias judgement** | | | **Low** | The study was registered in a clinical trial registry (IRCT20210224050481N1)​.There was no evidence of selective reporting, as all pre-specified outcomes appeared to be reported. |
| **Overall bias** | **Risk of bias judgement** | | | **Low** |  |
|  |  |  |  |  |  |
|  |  |  |  |  |  |
| **Unique ID** | 10 | **Study ID** | Reid 2007 | **Assessor** |  |
| **Ref or Label** |  | **Aim** | assignment to intervention (the 'intention-to-treat' effect) |  |  |
| **Experimental** |  | **Comparator** |  | **Source** |  |
| **Outcome** | Ankle ROM | **Results** |  | **Weight** | 1 |
| **Domain** | **Signalling question** | | | **Response** | **Comments** |
| **Bias arising from the randomization process** | 1.1 Was the allocation sequence random? | | | Y |  |
|  | 1.2 Was the allocation sequence concealed until participants were enrolled and assigned to interventions? | | | NI |  |
|  | 1.3 Did baseline differences between intervention groups suggest a problem with the randomization process? | | | N |  |
|  | **Risk of bias judgement** | | | **Some concerns** | The study used random assignment from a random numbers table to allocate participants to treatment groups. However, the method of allocation concealment was not explicitly stated, raising concerns about potential selection bias. |
| **Bias due to deviations from intended interventions** | 2.1.Were participants aware of their assigned intervention during the trial? | | | N |  |
|  | 2.2.Were carers and people delivering the interventions aware of participants' assigned intervention during the trial? | | | Y |  |
|  | 2.3. If Y/PY/NI to 2.1 or 2.2: Were there deviations from the intended intervention that arose because of the experimental context? | | | N |  |
|  | 2.4 If Y/PY to 2.3: Were these deviations likely to have affected the outcome? | | | NA |  |
|  | 2.5. If Y/PY/NI to 2.4: Were these deviations from intended intervention balanced between groups? | | | NA |  |
|  | 2.6 Was an appropriate analysis used to estimate the effect of assignment to intervention? | | | Y |  |
|  | 2.7 If N/PN/NI to 2.6: Was there potential for a substantial impact (on the result) of the failure to analyse participants in the group to which they were randomized? | | | NA |  |
|  | **Risk of bias judgement** | | | **Low** | The study was single-blinded, ensuring that outcome assessors were blinded to treatment allocation. Participants were not aware of whether they received the true mobilization or the sham treatment. However, the primary investigator (PI) delivering the intervention was not blinded, introducing potential bias. |
| **Bias due to missing outcome data** | 3.1 Were data for this outcome available for all, or nearly all, participants randomized? | | | Y |  |
|  | 3.2 If N/PN/NI to 3.1: Is there evidence that result was not biased by missing outcome data? | | | NA |  |
|  | 3.3 If N/PN to 3.2: Could missingness in the outcome depend on its true value? | | | NA |  |
|  | 3.4 If Y/PY/NI to 3.3: Is it likely that missingness in the outcome depended on its true value? | | | NA |  |
|  | **Risk of bias judgement** | | | **Low** | Only two participants (out of 25) dropped out, one due to an ankle re-sprain and another due to relocation. The dropout rate was minimal (8%), and the missing data were unlikely to impact the results significantly. |
| **Bias in measurement of the outcome** | 4.1 Was the method of measuring the outcome inappropriate? | | | N |  |
|  | 4.2 Could measurement or ascertainment of the outcome have differed between intervention groups? | | | N |  |
|  | 4.3 Were outcome assessors aware of the intervention received by study participants? | | | N |  |
|  | 4.4 If Y/PY/NI to 4.3: Could assessment of the outcome have been influenced by knowledge of intervention received? | | | NA |  |
|  | 4.5 If Y/PY/NI to 4.4: Is it likely that assessment of the outcome was influenced by knowledge of intervention received? | | | NA |  |
|  | **Risk of bias judgement** | | | **Low** | Outcome measurement was performed using a validated weight-bearing lunge test, ensuring objective assessment. The same examiner assessed dorsiflexion for each participant, maintaining measurement consistency. The assessors were blinded to group allocation, reducing the risk of detection bias. The ankle dorsiflexion range of motion (ROM) was measured using the Weight Bearing Lunge (WBL) test, which is an objective, tool-based assessment rather than a subjective patient-reported measure. |
| **Bias in selection of the reported result** | 5.1 Were the data that produced this result analysed in accordance with a pre-specified analysis plan that was finalized before unblinded outcome data were available for analysis? | | | NI |  |
|  | 5.2 ... multiple eligible outcome measurements (e.g. scales, definitions, time points) within the outcome domain? | | | N |  |
|  | 5.3 ... multiple eligible analyses of the data? | | | N |  |
|  | **Risk of bias judgement** | | | **Some concerns** | The study did not explicitly mention a pre-registered protocol. Although all expected outcomes were reported, the absence of a pre-specified statistical plan raises concerns about potential selective reporting. |
| **Overall bias** | **Risk of bias judgement** | | | **Some concerns** |  |
|  |  |  |  |  |  |
|  |  |  |  |  |  |
| **Unique ID** | 11 | **Study ID** | Gogate 2020 | **Assessor** |  |
| **Ref or Label** |  | **Aim** | assignment to intervention (the 'intention-to-treat' effect) |  |  |
| **Experimental** |  | **Comparator** |  | **Source** |  |
| **Outcome** | Y Balance test | **Results** |  | **Weight** | 1 |
| **Domain** | **Signalling question** | | | **Response** | **Comments** |
| **Bias arising from the randomization process** | 1.1 Was the allocation sequence random? | | | Y |  |
|  | 1.2 Was the allocation sequence concealed until participants were enrolled and assigned to interventions? | | | Y |  |
|  | 1.3 Did baseline differences between intervention groups suggest a problem with the randomization process? | | | N |  |
|  | **Risk of bias judgement** | | | **Low** |  |
| **Bias due to deviations from intended interventions** | 2.1.Were participants aware of their assigned intervention during the trial? | | | N |  |
|  | 2.2.Were carers and people delivering the interventions aware of participants' assigned intervention during the trial? | | | N |  |
|  | 2.3. If Y/PY/NI to 2.1 or 2.2: Were there deviations from the intended intervention that arose because of the experimental context? | | | NA |  |
|  | 2.4 If Y/PY to 2.3: Were these deviations likely to have affected the outcome? | | | NA |  |
|  | 2.5. If Y/PY/NI to 2.4: Were these deviations from intended intervention balanced between groups? | | | NA |  |
|  | 2.6 Was an appropriate analysis used to estimate the effect of assignment to intervention? | | | Y |  |
|  | 2.7 If N/PN/NI to 2.6: Was there potential for a substantial impact (on the result) of the failure to analyse participants in the group to which they were randomized? | | | NA |  |
|  | **Risk of bias judgement** | | | **Low** |  |
| **Bias due to missing outcome data** | 3.1 Were data for this outcome available for all, or nearly all, participants randomized? | | | Y |  |
|  | 3.2 If N/PN/NI to 3.1: Is there evidence that result was not biased by missing outcome data? | | | NA |  |
|  | 3.3 If N/PN to 3.2: Could missingness in the outcome depend on its true value? | | | NA |  |
|  | 3.4 If Y/PY/NI to 3.3: Is it likely that missingness in the outcome depended on its true value? | | | NA |  |
|  | **Risk of bias judgement** | | | **Low** |  |
| **Bias in measurement of the outcome** | 4.1 Was the method of measuring the outcome inappropriate? | | | N |  |
|  | 4.2 Could measurement or ascertainment of the outcome have differed between intervention groups? | | | N |  |
|  | 4.3 Were outcome assessors aware of the intervention received by study participants? | | | N |  |
|  | 4.4 If Y/PY/NI to 4.3: Could assessment of the outcome have been influenced by knowledge of intervention received? | | | NA |  |
|  | 4.5 If Y/PY/NI to 4.4: Is it likely that assessment of the outcome was influenced by knowledge of intervention received? | | | NA |  |
|  | **Risk of bias judgement** | | | **Low** | The YBT is a performance-based test rather than a self-reported measure. It does not rely on patient perception, memory, or subjective reporting, which reduces the risk of bias related to patient expectations or placebo effects. The test outcome is determined by a measured distance (cm) using a standardized tool, not by a subjective judgment of the assessor.This minimizes the risk of detection bias (where assessors might unconsciously influence results). |
| **Bias in selection of the reported result** | 5.1 Were the data that produced this result analysed in accordance with a pre-specified analysis plan that was finalized before unblinded outcome data were available for analysis? | | | NI |  |
|  | 5.2 ... multiple eligible outcome measurements (e.g. scales, definitions, time points) within the outcome domain? | | | N |  |
|  | 5.3 ... multiple eligible analyses of the data? | | | N |  |
|  | **Risk of bias judgement** | | | **Some concerns** |  |
| **Overall bias** | **Risk of bias judgement** | | | **Some concerns** |  |
|  |  |  |  |  |  |
|  |  |  |  |  |  |
| **Unique ID** | 12 | **Study ID** | Gogate 2020 | **Assessor** |  |
| **Ref or Label** |  | **Aim** | assignment to intervention (the 'intention-to-treat' effect) |  |  |
| **Experimental** |  | **Comparator** |  | **Source** |  |
| **Outcome** | Pain Pressure Threshold | **Results** |  | **Weight** | 1 |
| **Domain** | **Signalling question** | | | **Response** | **Comments** |
| **Bias arising from the randomization process** | 1.1 Was the allocation sequence random? | | | Y |  |
|  | 1.2 Was the allocation sequence concealed until participants were enrolled and assigned to interventions? | | | Y |  |
|  | 1.3 Did baseline differences between intervention groups suggest a problem with the randomization process? | | | N |  |
|  | **Risk of bias judgement** | | | **Low** |  |
| **Bias due to deviations from intended interventions** | 2.1.Were participants aware of their assigned intervention during the trial? | | | N |  |
|  | 2.2.Were carers and people delivering the interventions aware of participants' assigned intervention during the trial? | | | N |  |
|  | 2.3. If Y/PY/NI to 2.1 or 2.2: Were there deviations from the intended intervention that arose because of the experimental context? | | | NA |  |
|  | 2.4 If Y/PY to 2.3: Were these deviations likely to have affected the outcome? | | | NA |  |
|  | 2.5. If Y/PY/NI to 2.4: Were these deviations from intended intervention balanced between groups? | | | NA |  |
|  | 2.6 Was an appropriate analysis used to estimate the effect of assignment to intervention? | | | Y |  |
|  | 2.7 If N/PN/NI to 2.6: Was there potential for a substantial impact (on the result) of the failure to analyse participants in the group to which they were randomized? | | | NA |  |
|  | **Risk of bias judgement** | | | **Low** |  |
| **Bias due to missing outcome data** | 3.1 Were data for this outcome available for all, or nearly all, participants randomized? | | | Y |  |
|  | 3.2 If N/PN/NI to 3.1: Is there evidence that result was not biased by missing outcome data? | | | NA |  |
|  | 3.3 If N/PN to 3.2: Could missingness in the outcome depend on its true value? | | | NA |  |
|  | 3.4 If Y/PY/NI to 3.3: Is it likely that missingness in the outcome depended on its true value? | | | NA |  |
|  | **Risk of bias judgement** | | | **Low** |  |
| **Bias in measurement of the outcome** | 4.1 Was the method of measuring the outcome inappropriate? | | | N |  |
|  | 4.2 Could measurement or ascertainment of the outcome have differed between intervention groups? | | | N |  |
|  | 4.3 Were outcome assessors aware of the intervention received by study participants? | | | Y |  |
|  | 4.4 If Y/PY/NI to 4.3: Could assessment of the outcome have been influenced by knowledge of intervention received? | | | PY |  |
|  | 4.5 If Y/PY/NI to 4.4: Is it likely that assessment of the outcome was influenced by knowledge of intervention received? | | | PN |  |
|  | **Risk of bias judgement** | | | **Some concerns** | pain is a self reported outcome that could introduce bias even with blinding of the assessors. |
| **Bias in selection of the reported result** | 5.1 Were the data that produced this result analysed in accordance with a pre-specified analysis plan that was finalized before unblinded outcome data were available for analysis? | | | N |  |
|  | 5.2 ... multiple eligible outcome measurements (e.g. scales, definitions, time points) within the outcome domain? | | | N |  |
|  | 5.3 ... multiple eligible analyses of the data? | | | N |  |
|  | **Risk of bias judgement** | | | **Some concerns** |  |
| **Overall bias** | **Risk of bias judgement** | | | **Some concerns** |  |
|  |  |  |  |  |  |
|  |  |  |  |  |  |
| **Unique ID** | 13 | **Study ID** | Norouzi 2021 | **Assessor** |  |
| **Ref or Label** |  | **Aim** | assignment to intervention (the 'intention-to-treat' effect) |  |  |
| **Experimental** |  | **Comparator** |  | **Source** |  |
| **Outcome** | Pain intensity (VAS) | **Results** |  | **Weight** | 1 |
| **Domain** | **Signalling question** | | | **Response** | **Comments** |
| **Bias arising from the randomization process** | 1.1 Was the allocation sequence random? | | | Y |  |
|  | 1.2 Was the allocation sequence concealed until participants were enrolled and assigned to interventions? | | | Y |  |
|  | 1.3 Did baseline differences between intervention groups suggest a problem with the randomization process? | | | N |  |
|  | **Risk of bias judgement** | | | **Low** |  |
| **Bias due to deviations from intended interventions** | 2.1.Were participants aware of their assigned intervention during the trial? | | | N |  |
|  | 2.2.Were carers and people delivering the interventions aware of participants' assigned intervention during the trial? | | | Y |  |
|  | 2.3. If Y/PY/NI to 2.1 or 2.2: Were there deviations from the intended intervention that arose because of the experimental context? | | | N |  |
|  | 2.4 If Y/PY to 2.3: Were these deviations likely to have affected the outcome? | | | NA |  |
|  | 2.5. If Y/PY/NI to 2.4: Were these deviations from intended intervention balanced between groups? | | | NA |  |
|  | 2.6 Was an appropriate analysis used to estimate the effect of assignment to intervention? | | | Y |  |
|  | 2.7 If N/PN/NI to 2.6: Was there potential for a substantial impact (on the result) of the failure to analyse participants in the group to which they were randomized? | | | NA |  |
|  | **Risk of bias judgement** | | | **Low** |  |
| **Bias due to missing outcome data** | 3.1 Were data for this outcome available for all, or nearly all, participants randomized? | | | Y |  |
|  | 3.2 If N/PN/NI to 3.1: Is there evidence that result was not biased by missing outcome data? | | | NA |  |
|  | 3.3 If N/PN to 3.2: Could missingness in the outcome depend on its true value? | | | NA |  |
|  | 3.4 If Y/PY/NI to 3.3: Is it likely that missingness in the outcome depended on its true value? | | | NA |  |
|  | **Risk of bias judgement** | | | **Low** |  |
| **Bias in measurement of the outcome** | 4.1 Was the method of measuring the outcome inappropriate? | | | N |  |
|  | 4.2 Could measurement or ascertainment of the outcome have differed between intervention groups? | | | N |  |
|  | 4.3 Were outcome assessors aware of the intervention received by study participants? | | | Y |  |
|  | 4.4 If Y/PY/NI to 4.3: Could assessment of the outcome have been influenced by knowledge of intervention received? | | | PY |  |
|  | 4.5 If Y/PY/NI to 4.4: Is it likely that assessment of the outcome was influenced by knowledge of intervention received? | | | PN |  |
|  | **Risk of bias judgement** | | | **Some concerns** | Blinded assessors measured pain and ROM, but pain was self-reported (VAS scale), which may introduce bias. |
| **Bias in selection of the reported result** | 5.1 Were the data that produced this result analysed in accordance with a pre-specified analysis plan that was finalized before unblinded outcome data were available for analysis? | | | Y |  |
|  | 5.2 ... multiple eligible outcome measurements (e.g. scales, definitions, time points) within the outcome domain? | | | N |  |
|  | 5.3 ... multiple eligible analyses of the data? | | | N |  |
|  | **Risk of bias judgement** | | | **Low** |  |
| **Overall bias** | **Risk of bias judgement** | | | **Some concerns** |  |
|  |  |  |  |  |  |
|  |  |  |  |  |  |
| **Unique ID** | 14 | **Study ID** | Simsek 2018 | **Assessor** |  |
| **Ref or Label** |  | **Aim** | assignment to intervention (the 'intention-to-treat' effect) |  |  |
| **Experimental** |  | **Comparator** |  | **Source** |  |
| **Outcome** | Ankle ROM | **Results** |  | **Weight** | 1 |
| **Domain** | **Signalling question** | | | **Response** | **Comments** |
| **Bias arising from the randomization process** | 1.1 Was the allocation sequence random? | | | NI |  |
|  | 1.2 Was the allocation sequence concealed until participants were enrolled and assigned to interventions? | | | NI |  |
|  | 1.3 Did baseline differences between intervention groups suggest a problem with the randomization process? | | | N |  |
|  | **Risk of bias judgement** | | | **Some concerns** |  |
| **Bias due to deviations from intended interventions** | 2.1.Were participants aware of their assigned intervention during the trial? | | | PY |  |
|  | 2.2.Were carers and people delivering the interventions aware of participants' assigned intervention during the trial? | | | PY |  |
|  | 2.3. If Y/PY/NI to 2.1 or 2.2: Were there deviations from the intended intervention that arose because of the experimental context? | | | N |  |
|  | 2.4 If Y/PY to 2.3: Were these deviations likely to have affected the outcome? | | | NA |  |
|  | 2.5. If Y/PY/NI to 2.4: Were these deviations from intended intervention balanced between groups? | | | NA |  |
|  | 2.6 Was an appropriate analysis used to estimate the effect of assignment to intervention? | | | Y |  |
|  | 2.7 If N/PN/NI to 2.6: Was there potential for a substantial impact (on the result) of the failure to analyse participants in the group to which they were randomized? | | | NA |  |
|  | **Risk of bias judgement** | | | **Low** |  |
| **Bias due to missing outcome data** | 3.1 Were data for this outcome available for all, or nearly all, participants randomized? | | | Y |  |
|  | 3.2 If N/PN/NI to 3.1: Is there evidence that result was not biased by missing outcome data? | | | NA |  |
|  | 3.3 If N/PN to 3.2: Could missingness in the outcome depend on its true value? | | | NA |  |
|  | 3.4 If Y/PY/NI to 3.3: Is it likely that missingness in the outcome depended on its true value? | | | NA |  |
|  | **Risk of bias judgement** | | | **Low** |  |
| **Bias in measurement of the outcome** | 4.1 Was the method of measuring the outcome inappropriate? | | | N |  |
|  | 4.2 Could measurement or ascertainment of the outcome have differed between intervention groups? | | | N |  |
|  | 4.3 Were outcome assessors aware of the intervention received by study participants? | | | N |  |
|  | 4.4 If Y/PY/NI to 4.3: Could assessment of the outcome have been influenced by knowledge of intervention received? | | | NA |  |
|  | 4.5 If Y/PY/NI to 4.4: Is it likely that assessment of the outcome was influenced by knowledge of intervention received? | | | NA |  |
|  | **Risk of bias judgement** | | | **Low** | The ankle dorsiflexion range of motion (ROM) was measured using the Weight Bearing Lunge (WBL) test, which is an objective, tool-based assessment rather than a subjective patient-reported measure. |
| **Bias in selection of the reported result** | 5.1 Were the data that produced this result analysed in accordance with a pre-specified analysis plan that was finalized before unblinded outcome data were available for analysis? | | | NI |  |
|  | 5.2 ... multiple eligible outcome measurements (e.g. scales, definitions, time points) within the outcome domain? | | | N |  |
|  | 5.3 ... multiple eligible analyses of the data? | | | N |  |
|  | **Risk of bias judgement** | | | **Some concerns** |  |
| **Overall bias** | **Risk of bias judgement** | | | **Some concerns** |  |
|  |  |  |  |  |  |
|  |  |  |  |  |  |
| **Unique ID** | 15 | **Study ID** | Simsek 2018 | **Assessor** |  |
| **Ref or Label** |  | **Aim** | assignment to intervention (the 'intention-to-treat' effect) |  |  |
| **Experimental** |  | **Comparator** |  | **Source** |  |
| **Outcome** | SEBT | **Results** |  | **Weight** | 1 |
| **Domain** | **Signalling question** | | | **Response** | **Comments** |
| **Bias arising from the randomization process** | 1.1 Was the allocation sequence random? | | | NI |  |
|  | 1.2 Was the allocation sequence concealed until participants were enrolled and assigned to interventions? | | | NI |  |
|  | 1.3 Did baseline differences between intervention groups suggest a problem with the randomization process? | | | N |  |
|  | **Risk of bias judgement** | | | **Some concerns** |  |
| **Bias due to deviations from intended interventions** | 2.1.Were participants aware of their assigned intervention during the trial? | | | PY |  |
|  | 2.2.Were carers and people delivering the interventions aware of participants' assigned intervention during the trial? | | | PY |  |
|  | 2.3. If Y/PY/NI to 2.1 or 2.2: Were there deviations from the intended intervention that arose because of the experimental context? | | | N |  |
|  | 2.4 If Y/PY to 2.3: Were these deviations likely to have affected the outcome? | | | NA |  |
|  | 2.5. If Y/PY/NI to 2.4: Were these deviations from intended intervention balanced between groups? | | | NA |  |
|  | 2.6 Was an appropriate analysis used to estimate the effect of assignment to intervention? | | | Y |  |
|  | 2.7 If N/PN/NI to 2.6: Was there potential for a substantial impact (on the result) of the failure to analyse participants in the group to which they were randomized? | | | NA |  |
|  | **Risk of bias judgement** | | | **Low** |  |
| **Bias due to missing outcome data** | 3.1 Were data for this outcome available for all, or nearly all, participants randomized? | | | Y |  |
|  | 3.2 If N/PN/NI to 3.1: Is there evidence that result was not biased by missing outcome data? | | | NA |  |
|  | 3.3 If N/PN to 3.2: Could missingness in the outcome depend on its true value? | | | NA |  |
|  | 3.4 If Y/PY/NI to 3.3: Is it likely that missingness in the outcome depended on its true value? | | | NA |  |
|  | **Risk of bias judgement** | | | **Low** |  |
| **Bias in measurement of the outcome** | 4.1 Was the method of measuring the outcome inappropriate? | | | N |  |
|  | 4.2 Could measurement or ascertainment of the outcome have differed between intervention groups? | | | N |  |
|  | 4.3 Were outcome assessors aware of the intervention received by study participants? | | | PY |  |
|  | 4.4 If Y/PY/NI to 4.3: Could assessment of the outcome have been influenced by knowledge of intervention received? | | | N |  |
|  | 4.5 If Y/PY/NI to 4.4: Is it likely that assessment of the outcome was influenced by knowledge of intervention received? | | | NA |  |
|  | **Risk of bias judgement** | | | **Low** | The Star Excursion Balance Test (SEBT) is an objective, tool-based measure of dynamic balance rather than a subjective patient-reported outcome. It provides quantifiable data on reach distances in multiple directions, making it a reliable and reproducible assessment of balance control. |
| **Bias in selection of the reported result** | 5.1 Were the data that produced this result analysed in accordance with a pre-specified analysis plan that was finalized before unblinded outcome data were available for analysis? | | | NI |  |
|  | 5.2 ... multiple eligible outcome measurements (e.g. scales, definitions, time points) within the outcome domain? | | | N |  |
|  | 5.3 ... multiple eligible analyses of the data? | | | N |  |
|  | **Risk of bias judgement** | | | **Some concerns** |  |
| **Overall bias** | **Risk of bias judgement** | | | **Some concerns** |  |
|  |  |  |  |  |  |
|  |  |  |  |  |  |
| **Unique ID** | 16 | **Study ID** | Nguyen 2020 | **Assessor** |  |
| **Ref or Label** |  | **Aim** | assignment to intervention (the 'intention-to-treat' effect) |  |  |
| **Experimental** |  | **Comparator** |  | **Source** |  |
| **Outcome** | Pain intensity (VAS) | **Results** |  | **Weight** | 1 |
| **Domain** | **Signalling question** | | | **Response** | **Comments** |
| **Bias arising from the randomization process** | 1.1 Was the allocation sequence random? | | | Y |  |
|  | 1.2 Was the allocation sequence concealed until participants were enrolled and assigned to interventions? | | | Y |  |
|  | 1.3 Did baseline differences between intervention groups suggest a problem with the randomization process? | | | N |  |
|  | **Risk of bias judgement** | | | **Low** |  |
| **Bias due to deviations from intended interventions** | 2.1.Were participants aware of their assigned intervention during the trial? | | | N |  |
|  | 2.2.Were carers and people delivering the interventions aware of participants' assigned intervention during the trial? | | | Y |  |
|  | 2.3. If Y/PY/NI to 2.1 or 2.2: Were there deviations from the intended intervention that arose because of the experimental context? | | | N |  |
|  | 2.4 If Y/PY to 2.3: Were these deviations likely to have affected the outcome? | | | NA |  |
|  | 2.5. If Y/PY/NI to 2.4: Were these deviations from intended intervention balanced between groups? | | | NA |  |
|  | 2.6 Was an appropriate analysis used to estimate the effect of assignment to intervention? | | | Y |  |
|  | 2.7 If N/PN/NI to 2.6: Was there potential for a substantial impact (on the result) of the failure to analyse participants in the group to which they were randomized? | | | NA |  |
|  | **Risk of bias judgement** | | | **Low** |  |
| **Bias due to missing outcome data** | 3.1 Were data for this outcome available for all, or nearly all, participants randomized? | | | Y |  |
|  | 3.2 If N/PN/NI to 3.1: Is there evidence that result was not biased by missing outcome data? | | | NA |  |
|  | 3.3 If N/PN to 3.2: Could missingness in the outcome depend on its true value? | | | NA |  |
|  | 3.4 If Y/PY/NI to 3.3: Is it likely that missingness in the outcome depended on its true value? | | | NA |  |
|  | **Risk of bias judgement** | | | **Low** |  |
| **Bias in measurement of the outcome** | 4.1 Was the method of measuring the outcome inappropriate? | | | N |  |
|  | 4.2 Could measurement or ascertainment of the outcome have differed between intervention groups? | | | N |  |
|  | 4.3 Were outcome assessors aware of the intervention received by study participants? | | | PY |  |
|  | 4.4 If Y/PY/NI to 4.3: Could assessment of the outcome have been influenced by knowledge of intervention received? | | | PY |  |
|  | 4.5 If Y/PY/NI to 4.4: Is it likely that assessment of the outcome was influenced by knowledge of intervention received? | | | N |  |
|  | **Risk of bias judgement** | | | **Some concerns** | The Visual Analogue Scale (VAS) for pain assessment is a subjective, patient-dependent measure rather than an objective tool-based outcome. Unlike tests that rely on quantifiable physical measurements, the VAS score is entirely based on the patient’s personal perception of pain, which introduces potential bias and variability. |
| **Bias in selection of the reported result** | 5.1 Were the data that produced this result analysed in accordance with a pre-specified analysis plan that was finalized before unblinded outcome data were available for analysis? | | | NI |  |
|  | 5.2 ... multiple eligible outcome measurements (e.g. scales, definitions, time points) within the outcome domain? | | | N |  |
|  | 5.3 ... multiple eligible analyses of the data? | | | N |  |
|  | **Risk of bias judgement** | | | **Some concerns** |  |
| **Overall bias** | **Risk of bias judgement** | | | **Some concerns** |  |
|  |  |  |  |  |  |
|  |  |  |  |  |  |
| **Unique ID** | 17 | **Study ID** | Nguyen 2020 | **Assessor** |  |
| **Ref or Label** |  | **Aim** | assignment to intervention (the 'intention-to-treat' effect) |  |  |
| **Experimental** |  | **Comparator** |  | **Source** |  |
| **Outcome** | Stiffness perception | **Results** |  | **Weight** | 1 |
| **Domain** | **Signalling question** | | | **Response** | **Comments** |
| **Bias arising from the randomization process** | 1.1 Was the allocation sequence random? | | | Y |  |
|  | 1.2 Was the allocation sequence concealed until participants were enrolled and assigned to interventions? | | | Y |  |
|  | 1.3 Did baseline differences between intervention groups suggest a problem with the randomization process? | | | N |  |
|  | **Risk of bias judgement** | | | **Low** |  |
| **Bias due to deviations from intended interventions** | 2.1.Were participants aware of their assigned intervention during the trial? | | | N |  |
|  | 2.2.Were carers and people delivering the interventions aware of participants' assigned intervention during the trial? | | | Y |  |
|  | 2.3. If Y/PY/NI to 2.1 or 2.2: Were there deviations from the intended intervention that arose because of the experimental context? | | | N |  |
|  | 2.4 If Y/PY to 2.3: Were these deviations likely to have affected the outcome? | | | NA |  |
|  | 2.5. If Y/PY/NI to 2.4: Were these deviations from intended intervention balanced between groups? | | | NA |  |
|  | 2.6 Was an appropriate analysis used to estimate the effect of assignment to intervention? | | | Y |  |
|  | 2.7 If N/PN/NI to 2.6: Was there potential for a substantial impact (on the result) of the failure to analyse participants in the group to which they were randomized? | | | NA |  |
|  | **Risk of bias judgement** | | | **Low** |  |
| **Bias due to missing outcome data** | 3.1 Were data for this outcome available for all, or nearly all, participants randomized? | | | Y |  |
|  | 3.2 If N/PN/NI to 3.1: Is there evidence that result was not biased by missing outcome data? | | | NA |  |
|  | 3.3 If N/PN to 3.2: Could missingness in the outcome depend on its true value? | | | NA |  |
|  | 3.4 If Y/PY/NI to 3.3: Is it likely that missingness in the outcome depended on its true value? | | | NA |  |
|  | **Risk of bias judgement** | | | **Low** |  |
| **Bias in measurement of the outcome** | 4.1 Was the method of measuring the outcome inappropriate? | | | N |  |
|  | 4.2 Could measurement or ascertainment of the outcome have differed between intervention groups? | | | N |  |
|  | 4.3 Were outcome assessors aware of the intervention received by study participants? | | | PY |  |
|  | 4.4 If Y/PY/NI to 4.3: Could assessment of the outcome have been influenced by knowledge of intervention received? | | | PY |  |
|  | 4.5 If Y/PY/NI to 4.4: Is it likely that assessment of the outcome was influenced by knowledge of intervention received? | | | PN |  |
|  | **Risk of bias judgement** | | | **Some concerns** | The stiffness perception assessment using the Visual Analogue Scale (VAS) is a subjective, patient-dependent measure, meaning it relies entirely on the individual's personal perception rather than an objective, tool-based assessment. Unlike standardized biomechanical measurements, this outcome is prone to variability, bias, and individual interpretation differences. |
| **Bias in selection of the reported result** | 5.1 Were the data that produced this result analysed in accordance with a pre-specified analysis plan that was finalized before unblinded outcome data were available for analysis? | | | NI |  |
|  | 5.2 ... multiple eligible outcome measurements (e.g. scales, definitions, time points) within the outcome domain? | | | N |  |
|  | 5.3 ... multiple eligible analyses of the data? | | | N |  |
|  | **Risk of bias judgement** | | | **Some concerns** |  |
| **Overall bias** | **Risk of bias judgement** | | | **Some concerns** |  |
|  |  |  |  |  |  |
|  |  |  |  |  |  |
| **Unique ID** | 18 | **Study ID** | Nguyen 2021 | **Assessor** |  |
| **Ref or Label** |  | **Aim** | assignment to intervention (the 'intention-to-treat' effect) |  |  |
| **Experimental** |  | **Comparator** |  | **Source** |  |
| **Outcome** | Y Balance test | **Results** |  | **Weight** | 1 |
| **Domain** | **Signalling question** | | | **Response** | **Comments** |
| **Bias arising from the randomization process** | 1.1 Was the allocation sequence random? | | | Y |  |
|  | 1.2 Was the allocation sequence concealed until participants were enrolled and assigned to interventions? | | | NI |  |
|  | 1.3 Did baseline differences between intervention groups suggest a problem with the randomization process? | | | N |  |
|  | **Risk of bias judgement** | | | **Some concerns** |  |
| **Bias due to deviations from intended interventions** | 2.1.Were participants aware of their assigned intervention during the trial? | | | N |  |
|  | 2.2.Were carers and people delivering the interventions aware of participants' assigned intervention during the trial? | | | Y |  |
|  | 2.3. If Y/PY/NI to 2.1 or 2.2: Were there deviations from the intended intervention that arose because of the experimental context? | | | N |  |
|  | 2.4 If Y/PY to 2.3: Were these deviations likely to have affected the outcome? | | | NA |  |
|  | 2.5. If Y/PY/NI to 2.4: Were these deviations from intended intervention balanced between groups? | | | NA |  |
|  | 2.6 Was an appropriate analysis used to estimate the effect of assignment to intervention? | | | Y |  |
|  | 2.7 If N/PN/NI to 2.6: Was there potential for a substantial impact (on the result) of the failure to analyse participants in the group to which they were randomized? | | | NA |  |
|  | **Risk of bias judgement** | | | **Low** |  |
| **Bias due to missing outcome data** | 3.1 Were data for this outcome available for all, or nearly all, participants randomized? | | | Y |  |
|  | 3.2 If N/PN/NI to 3.1: Is there evidence that result was not biased by missing outcome data? | | | NA |  |
|  | 3.3 If N/PN to 3.2: Could missingness in the outcome depend on its true value? | | | NA |  |
|  | 3.4 If Y/PY/NI to 3.3: Is it likely that missingness in the outcome depended on its true value? | | | NA |  |
|  | **Risk of bias judgement** | | | **Low** |  |
| **Bias in measurement of the outcome** | 4.1 Was the method of measuring the outcome inappropriate? | | | N |  |
|  | 4.2 Could measurement or ascertainment of the outcome have differed between intervention groups? | | | N |  |
|  | 4.3 Were outcome assessors aware of the intervention received by study participants? | | | N |  |
|  | 4.4 If Y/PY/NI to 4.3: Could assessment of the outcome have been influenced by knowledge of intervention received? | | | NA |  |
|  | 4.5 If Y/PY/NI to 4.4: Is it likely that assessment of the outcome was influenced by knowledge of intervention received? | | | NA |  |
|  | **Risk of bias judgement** | | | **Low** | YBT is an objective, tool-based measure with high reliability and minimal influence from subjective factors. |
| **Bias in selection of the reported result** | 5.1 Were the data that produced this result analysed in accordance with a pre-specified analysis plan that was finalized before unblinded outcome data were available for analysis? | | | Y |  |
|  | 5.2 ... multiple eligible outcome measurements (e.g. scales, definitions, time points) within the outcome domain? | | | N |  |
|  | 5.3 ... multiple eligible analyses of the data? | | | N |  |
|  | **Risk of bias judgement** | | | **Low** |  |
| **Overall bias** | **Risk of bias judgement** | | | **Some concerns** |  |
|  |  |  |  |  |  |
|  |  |  |  |  |  |
| **Unique ID** | 19 | **Study ID** | Nguyen 2021 | **Assessor** |  |
| **Ref or Label** |  | **Aim** | assignment to intervention (the 'intention-to-treat' effect) |  |  |
| **Experimental** |  | **Comparator** |  | **Source** |  |
| **Outcome** | Pain intensity (VAS) | **Results** |  | **Weight** | 1 |
| **Domain** | **Signalling question** | | | **Response** | **Comments** |
| **Bias arising from the randomization process** | 1.1 Was the allocation sequence random? | | | Y |  |
|  | 1.2 Was the allocation sequence concealed until participants were enrolled and assigned to interventions? | | | NI |  |
|  | 1.3 Did baseline differences between intervention groups suggest a problem with the randomization process? | | | N |  |
|  | **Risk of bias judgement** | | | **Some concerns** |  |
| **Bias due to deviations from intended interventions** | 2.1.Were participants aware of their assigned intervention during the trial? | | | N |  |
|  | 2.2.Were carers and people delivering the interventions aware of participants' assigned intervention during the trial? | | | Y |  |
|  | 2.3. If Y/PY/NI to 2.1 or 2.2: Were there deviations from the intended intervention that arose because of the experimental context? | | | N |  |
|  | 2.4 If Y/PY to 2.3: Were these deviations likely to have affected the outcome? | | | NA |  |
|  | 2.5. If Y/PY/NI to 2.4: Were these deviations from intended intervention balanced between groups? | | | NA |  |
|  | 2.6 Was an appropriate analysis used to estimate the effect of assignment to intervention? | | | Y |  |
|  | 2.7 If N/PN/NI to 2.6: Was there potential for a substantial impact (on the result) of the failure to analyse participants in the group to which they were randomized? | | | NA |  |
|  | **Risk of bias judgement** | | | **Low** |  |
| **Bias due to missing outcome data** | 3.1 Were data for this outcome available for all, or nearly all, participants randomized? | | | Y |  |
|  | 3.2 If N/PN/NI to 3.1: Is there evidence that result was not biased by missing outcome data? | | | NA |  |
|  | 3.3 If N/PN to 3.2: Could missingness in the outcome depend on its true value? | | | NA |  |
|  | 3.4 If Y/PY/NI to 3.3: Is it likely that missingness in the outcome depended on its true value? | | | NA |  |
|  | **Risk of bias judgement** | | | **Low** |  |
| **Bias in measurement of the outcome** | 4.1 Was the method of measuring the outcome inappropriate? | | | N |  |
|  | 4.2 Could measurement or ascertainment of the outcome have differed between intervention groups? | | | N |  |
|  | 4.3 Were outcome assessors aware of the intervention received by study participants? | | | PY |  |
|  | 4.4 If Y/PY/NI to 4.3: Could assessment of the outcome have been influenced by knowledge of intervention received? | | | PY |  |
|  | 4.5 If Y/PY/NI to 4.4: Is it likely that assessment of the outcome was influenced by knowledge of intervention received? | | | PN |  |
|  | **Risk of bias judgement** | | | **Some concerns** | The Visual Analogue Scale (VAS) for pain assessment is a subjective, patient-dependent measure rather than an objective tool-based outcome. |
| **Bias in selection of the reported result** | 5.1 Were the data that produced this result analysed in accordance with a pre-specified analysis plan that was finalized before unblinded outcome data were available for analysis? | | | Y |  |
|  | 5.2 ... multiple eligible outcome measurements (e.g. scales, definitions, time points) within the outcome domain? | | | N |  |
|  | 5.3 ... multiple eligible analyses of the data? | | | N |  |
|  | **Risk of bias judgement** | | | **Low** |  |
| **Overall bias** | **Risk of bias judgement** | | | **Some concerns** |  |
|  |  |  |  |  |  |
|  |  |  |  |  |  |
| **Unique ID** | 20 | **Study ID** | Nguyen 2021 | **Assessor** |  |
| **Ref or Label** |  | **Aim** | assignment to intervention (the 'intention-to-treat' effect) |  |  |
| **Experimental** |  | **Comparator** |  | **Source** |  |
| **Outcome** | Stiffness Perception | **Results** |  | **Weight** | 1 |
| **Domain** | **Signalling question** | | | **Response** | **Comments** |
| **Bias arising from the randomization process** | 1.1 Was the allocation sequence random? | | | Y |  |
|  | 1.2 Was the allocation sequence concealed until participants were enrolled and assigned to interventions? | | | NI |  |
|  | 1.3 Did baseline differences between intervention groups suggest a problem with the randomization process? | | | N |  |
|  | **Risk of bias judgement** | | | **Some concerns** |  |
| **Bias due to deviations from intended interventions** | 2.1.Were participants aware of their assigned intervention during the trial? | | | N |  |
|  | 2.2.Were carers and people delivering the interventions aware of participants' assigned intervention during the trial? | | | Y |  |
|  | 2.3. If Y/PY/NI to 2.1 or 2.2: Were there deviations from the intended intervention that arose because of the experimental context? | | | N |  |
|  | 2.4 If Y/PY to 2.3: Were these deviations likely to have affected the outcome? | | | NA |  |
|  | 2.5. If Y/PY/NI to 2.4: Were these deviations from intended intervention balanced between groups? | | | NA |  |
|  | 2.6 Was an appropriate analysis used to estimate the effect of assignment to intervention? | | | Y |  |
|  | 2.7 If N/PN/NI to 2.6: Was there potential for a substantial impact (on the result) of the failure to analyse participants in the group to which they were randomized? | | | NA |  |
|  | **Risk of bias judgement** | | | **Low** |  |
| **Bias due to missing outcome data** | 3.1 Were data for this outcome available for all, or nearly all, participants randomized? | | | Y |  |
|  | 3.2 If N/PN/NI to 3.1: Is there evidence that result was not biased by missing outcome data? | | | NA |  |
|  | 3.3 If N/PN to 3.2: Could missingness in the outcome depend on its true value? | | | NA |  |
|  | 3.4 If Y/PY/NI to 3.3: Is it likely that missingness in the outcome depended on its true value? | | | NA |  |
|  | **Risk of bias judgement** | | | **Low** |  |
| **Bias in measurement of the outcome** | 4.1 Was the method of measuring the outcome inappropriate? | | | N |  |
|  | 4.2 Could measurement or ascertainment of the outcome have differed between intervention groups? | | | N |  |
|  | 4.3 Were outcome assessors aware of the intervention received by study participants? | | | PY |  |
|  | 4.4 If Y/PY/NI to 4.3: Could assessment of the outcome have been influenced by knowledge of intervention received? | | | PY |  |
|  | 4.5 If Y/PY/NI to 4.4: Is it likely that assessment of the outcome was influenced by knowledge of intervention received? | | | PN |  |
|  | **Risk of bias judgement** | | | **Some concerns** | Since stiffness perception is entirely patient-reported, it is a subjective measure influenced by psychological, emotional, and individual factors. |
| **Bias in selection of the reported result** | 5.1 Were the data that produced this result analysed in accordance with a pre-specified analysis plan that was finalized before unblinded outcome data were available for analysis? | | | Y |  |
|  | 5.2 ... multiple eligible outcome measurements (e.g. scales, definitions, time points) within the outcome domain? | | | N |  |
|  | 5.3 ... multiple eligible analyses of the data? | | | N |  |
|  | **Risk of bias judgement** | | | **Low** |  |
| **Overall bias** | **Risk of bias judgement** | | | **Some concerns** |  |
|  |  |  |  |  |  |
|  |  |  |  |  |  |
| **Unique ID** | 21 | **Study ID** | Collins 2004 | **Assessor** |  |
| **Ref or Label** |  | **Aim** | assignment to intervention (the 'intention-to-treat' effect) |  |  |
| **Experimental** |  | **Comparator** |  | **Source** |  |
| **Outcome** | Pain presuure threshold | **Results** |  | **Weight** | 1 |
| **Domain** | **Signalling question** | | | **Response** | **Comments** |
| **Bias arising from the randomization process** | 1.1 Was the allocation sequence random? | | | NI |  |
|  | 1.2 Was the allocation sequence concealed until participants were enrolled and assigned to interventions? | | | NI |  |
|  | 1.3 Did baseline differences between intervention groups suggest a problem with the randomization process? | | | N |  |
|  | **Risk of bias judgement** | | | **Some concerns** |  |
| **Bias due to deviations from intended interventions** | 2.1.Were participants aware of their assigned intervention during the trial? | | | N |  |
|  | 2.2.Were carers and people delivering the interventions aware of participants' assigned intervention during the trial? | | | N |  |
|  | 2.3. If Y/PY/NI to 2.1 or 2.2: Were there deviations from the intended intervention that arose because of the experimental context? | | | NA |  |
|  | 2.4 If Y/PY to 2.3: Were these deviations likely to have affected the outcome? | | | NA |  |
|  | 2.5. If Y/PY/NI to 2.4: Were these deviations from intended intervention balanced between groups? | | | NA |  |
|  | 2.6 Was an appropriate analysis used to estimate the effect of assignment to intervention? | | | Y |  |
|  | 2.7 If N/PN/NI to 2.6: Was there potential for a substantial impact (on the result) of the failure to analyse participants in the group to which they were randomized? | | | NA |  |
|  | **Risk of bias judgement** | | | **Low** |  |
| **Bias due to missing outcome data** | 3.1 Were data for this outcome available for all, or nearly all, participants randomized? | | | Y |  |
|  | 3.2 If N/PN/NI to 3.1: Is there evidence that result was not biased by missing outcome data? | | | NA |  |
|  | 3.3 If N/PN to 3.2: Could missingness in the outcome depend on its true value? | | | NA |  |
|  | 3.4 If Y/PY/NI to 3.3: Is it likely that missingness in the outcome depended on its true value? | | | NA |  |
|  | **Risk of bias judgement** | | | **Low** |  |
| **Bias in measurement of the outcome** | 4.1 Was the method of measuring the outcome inappropriate? | | | N |  |
|  | 4.2 Could measurement or ascertainment of the outcome have differed between intervention groups? | | | N |  |
|  | 4.3 Were outcome assessors aware of the intervention received by study participants? | | | PY |  |
|  | 4.4 If Y/PY/NI to 4.3: Could assessment of the outcome have been influenced by knowledge of intervention received? | | | PY |  |
|  | 4.5 If Y/PY/NI to 4.4: Is it likely that assessment of the outcome was influenced by knowledge of intervention received? | | | PN |  |
|  | **Risk of bias judgement** | | | **Some concerns** | Although pressure pain threshold (PPT) measurement using a digital pressure algometer appears objective because it quantifies pressure, it is still a subjective, patient-dependent measure. The outcome relies on the patient’s perception of pain and their voluntary response, making it prone to variability and bias. |
| **Bias in selection of the reported result** | 5.1 Were the data that produced this result analysed in accordance with a pre-specified analysis plan that was finalized before unblinded outcome data were available for analysis? | | | NI |  |
|  | 5.2 ... multiple eligible outcome measurements (e.g. scales, definitions, time points) within the outcome domain? | | | NI |  |
|  | 5.3 ... multiple eligible analyses of the data? | | | N |  |
|  | **Risk of bias judgement** | | | **Some concerns** |  |
| **Overall bias** | **Risk of bias judgement** | | | **Some concerns** |  |
|  |  |  |  |  |  |
|  |  |  |  |  |  |
| **Unique ID** | 22 | **Study ID** | Cruz-Diaz 2014 | **Assessor** |  |
| **Ref or Label** |  | **Aim** | assignment to intervention (the 'intention-to-treat' effect) |  |  |
| **Experimental** |  | **Comparator** |  | **Source** |  |
| **Outcome** | SEBT | **Results** |  | **Weight** | 1 |
| **Domain** | **Signalling question** | | | **Response** | **Comments** |
| **Bias arising from the randomization process** | 1.1 Was the allocation sequence random? | | | Y |  |
|  | 1.2 Was the allocation sequence concealed until participants were enrolled and assigned to interventions? | | | Y |  |
|  | 1.3 Did baseline differences between intervention groups suggest a problem with the randomization process? | | | N |  |
|  | **Risk of bias judgement** | | | **Low** |  |
| **Bias due to deviations from intended interventions** | 2.1.Were participants aware of their assigned intervention during the trial? | | | N |  |
|  | 2.2.Were carers and people delivering the interventions aware of participants' assigned intervention during the trial? | | | N |  |
|  | 2.3. If Y/PY/NI to 2.1 or 2.2: Were there deviations from the intended intervention that arose because of the experimental context? | | | NA |  |
|  | 2.4 If Y/PY to 2.3: Were these deviations likely to have affected the outcome? | | | NA |  |
|  | 2.5. If Y/PY/NI to 2.4: Were these deviations from intended intervention balanced between groups? | | | NA |  |
|  | 2.6 Was an appropriate analysis used to estimate the effect of assignment to intervention? | | | Y |  |
|  | 2.7 If N/PN/NI to 2.6: Was there potential for a substantial impact (on the result) of the failure to analyse participants in the group to which they were randomized? | | | NA |  |
|  | **Risk of bias judgement** | | | **Low** |  |
| **Bias due to missing outcome data** | 3.1 Were data for this outcome available for all, or nearly all, participants randomized? | | | Y |  |
|  | 3.2 If N/PN/NI to 3.1: Is there evidence that result was not biased by missing outcome data? | | | NA |  |
|  | 3.3 If N/PN to 3.2: Could missingness in the outcome depend on its true value? | | | NA |  |
|  | 3.4 If Y/PY/NI to 3.3: Is it likely that missingness in the outcome depended on its true value? | | | NA |  |
|  | **Risk of bias judgement** | | | **Low** |  |
| **Bias in measurement of the outcome** | 4.1 Was the method of measuring the outcome inappropriate? | | | N |  |
|  | 4.2 Could measurement or ascertainment of the outcome have differed between intervention groups? | | | N |  |
|  | 4.3 Were outcome assessors aware of the intervention received by study participants? | | | N |  |
|  | 4.4 If Y/PY/NI to 4.3: Could assessment of the outcome have been influenced by knowledge of intervention received? | | | NA |  |
|  | 4.5 If Y/PY/NI to 4.4: Is it likely that assessment of the outcome was influenced by knowledge of intervention received? | | | NA |  |
|  | **Risk of bias judgement** | | | **Low** | The Star Excursion Balance Test (SEBT) is an objective, tool-based measure of dynamic balance rather than a subjective patient-reported outcome. It provides quantifiable data on reach distances in multiple directions, making it a reliable and reproducible assessment of balance control. |
| **Bias in selection of the reported result** | 5.1 Were the data that produced this result analysed in accordance with a pre-specified analysis plan that was finalized before unblinded outcome data were available for analysis? | | | NI |  |
|  | 5.2 ... multiple eligible outcome measurements (e.g. scales, definitions, time points) within the outcome domain? | | | N |  |
|  | 5.3 ... multiple eligible analyses of the data? | | | N |  |
|  | **Risk of bias judgement** | | | **Some concerns** |  |
| **Overall bias** | **Risk of bias judgement** | | | **Some concerns** |  |
